# Chemical characterization, pathway enrichments and bioactive potentials of catechin-producing endophytic fungi isolated from tea leaves†

**DOI:** 10.1039/d4ra05758a

**Published:** 2024-10-21

**Authors:** Dwinder Sidhu, M. Vasundhara, Priyankar Dey

**Affiliations:** a Department of Biotechnology, Thapar Institute of Engineering & Technology Patiala Punjab 147004 India priyankar.dey@thapar.edu mvasundhara@thapar.edu +91-9064275660 +91-8146480908

## Abstract

Endophytes acquire flavonoid biosynthetic genes from the host medicinal plants. Despite tea (*Camellia sinensis* (L.) Kuntze) being the major source of bioactive catechins, catechin-producing endophytic fungi have never been reported from the tea plant. Here, we report the isolation and characterization of catechin-producing endophytic fungi isolated from tea leaves, their chemical characterization, and associated bioactivities. Among the nine isolated endophytes, two (CSPL6 and CSPL5b) produced catechin (381.48 and 166.40 μg per mg extract) and epigallocatechin-*o*-gallate (EGCG; 484.41 and 281.99 μg per mg extract) as quantified by high-performance liquid chromatography (HPLC). The isolates were identified as *Pseudopestalotiopsis camelliae-sinensis* and *Didymella sinensis* based on molecular and morphological characterization. Untargeted metabolomics using gas-chromatography mass spectroscopy (GCMS) revealed the presence of several bioactive phytochemicals mostly belonging to tyrosols, pyridoxines, fatty acids, aminopyrimidine, and benzenetriol classes. Metabolic pathways pertaining to the biosynthesis of unsaturated fatty acids (UFAs), butanoate metabolism, and linoleic acid metabolism were highly enriched in both catechin-producing isolates. The isolates were able to differentially scavenge intracellular O_2_ and N_2_ free-radicals, but CSPL5b demonstrated relatively superior bioactivities compared to CSPL6. Both isolates stimulated the growth of various probiotic strains, indicating prebiotic effects that are otherwise known to be associated with catechins. Collectively, the current study demonstrated that fungal endophytes CSPL6 and CSPL5b, isolated from tea leaves, could be used as alternative sources of catechins, and hold promising potential in evidence-based therapeutics.

## Abstract 2

Endophytes acquire flavonoid biosynthetic genes from the host medicinal plants.

## Graphical abstract

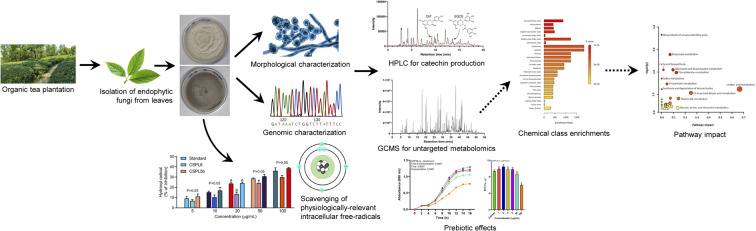

## Introduction

Environmental elements (*e.g.*, temperature, light, moisture, soil composition) substantially impact the quality and quantity of the bioactive phytochemical content of medicinal plants. Endophytes, which often share similar biosynthetic pathways with their host plants, have increasingly been recognized for their impact on stress tolerance and the metabolomics output of medicinal plants.^1,2^ Establishing enduring and mutually beneficial associations between host plants and endophytes enhances plant growth and proves particularly advantageous in agricultural applications. Thus, with climate change impacting the agricultural output of industrially relevant medicinal plants, there is an urgent need for alternative sources of pharmacologically important phytochemicals. Indeed, this is more relevant in the case of medicinal plants of the Himalayan region which is gravely impacted by climate change.^3,4^

*C. sinensis* (L.) Kuntze, commonly known as tea, is one of the major cash crops of the sub-Himalayan region for its use as a recreational drink and functional food, as well as in evidence-based pharmacology. Catechins, the major bioactive component in tea, are attributed to metabolic health benefits through anti-inflammatory and antioxidative effects.^5,6^ However, due to the global increased demand for catechins in the nutraceutical, pharma, and cosmetic industries^7^ and the coinciding decline in tea production,^8^ there has been increased demand for alternate sources of catechins. In fact, this is further triggered by global climate change, which negatively impacts the quality and quantity of tea production globally.^8–12^ Owing to the intricate and dynamic plant–microbe interaction, a variety of phytochemical synthesizing genes are horizontally transferred to the endophytic microbes that often serve as alternative and better sources of medicinally important metabolites. Therefore, tea-derived endophytes are the preferred alternative source for the sustainable production of bioactive catechins. To date, various endophytes have been isolated from *C. sinensis*, demonstrating diverse bioactivities,^13–15^ and catechin-producing endophytes have been reported from other plant sources.^16–19^ While it is imperative to attempt isolation of catechin-producing endophytes from *C. sinensis*, the major natural source of catechins, to our knowledge, this has never been reported before. Indeed, this could be because chances of identifying endophytes with desired characteristics are influenced by counterintuitive agricultural practices where agrochemicals deplete the microbes that otherwise maintain commensalism with the tea plant.

Therefore, in the present study, we attempted to isolate and characterize catechin-producing endophytic fungi from the leaves of organically grown tea plants. Catechin and epigallocatechin-*o*-gallate (EGCG) were quantified using high-performance liquid chromatography (HPLC) and untargeted metabolomics fingerprinting of the isolates was performed by gas chromatography-mass spectroscopy (GCMS). The major metabolic pathways were identified based on the identified metabolites. The bioactivities of the chemically characterized extracts were tested by scavenging the major intracellular free-radicals, and the impact of the extracts was tested for possible prebiotic effects on various *Lactobacillus* spp. Therefore, for the first time, catechin-producing endophytes were isolated as an alternative to *C. sinensis*, and chemical characterization and bioactivity evaluation were undertaken.

## Materials and methods

### Sample collection and isolation of endophytes

**Fig. 1:**
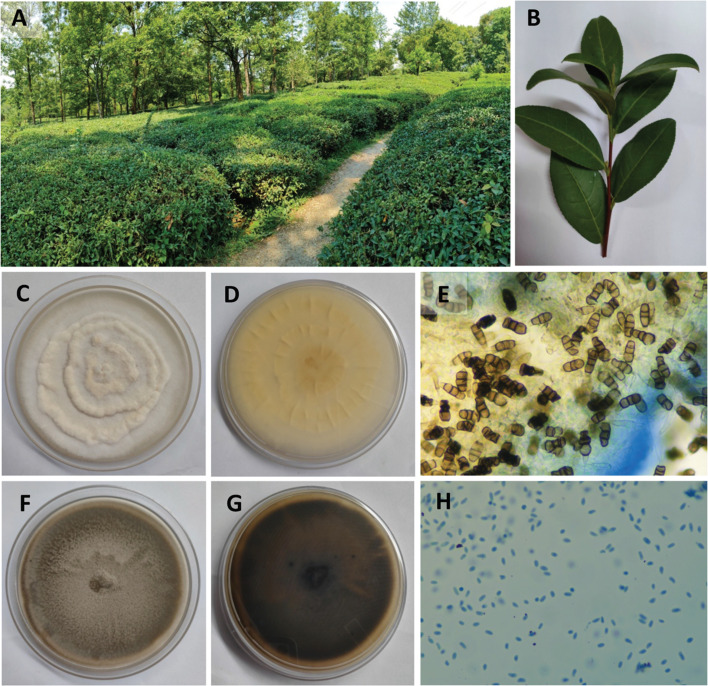
(A and B) Tea plantation in Himachal Pradesh (C–E) morphological and microscopic features of endophytic fungi CSPL6: front view of CSPL6 on PDA plate, back view of CSPL6 on PDA plate, and spores of CSPL6 under the microscope (F–H) morphological and microscopic features of endophytic fungi CSPL5b: front view of CSPL5b on PDA plate, back view of CSPL5b on PDA plate, and spores of CSPL5b under the microscope.

The study was approved by the Institutional Biosafety Committee (TIET/IBSC/23-24/05). The fresh and disease-free leaves of *C. sinensis* were collected in June 2022 at a domestic organic tea garden in Palampur, Himachal Pradesh, India (Fig. 1A and B, latitude: 32.115198/N 32° 6′ 54.712′′; longitude: 76.558635/E 76° 33′ 31.087′′). The leaves were packed in sterile polyethylene bags and stored at 4 °C until further use. Plant material was authenticated and was stored with an authentication number BTD/TIET/22/04. Endophytic fungi were isolated from the leaves following standard protocol with slight modifications.^20,21^ In brief, the leaves were washed with double distilled water twice for the removal of dirt from the surface. Leaves were then chopped into 1 × 1 cm pieces and then sequentially sterilized using 70% ethanol (v/v) for 60 s, 2–4% sodium hypochlorite (NaOCl; v/v) for 1.5 min, and finally twice with sterile distilled water. The upper layer of the leaves was carefully removed using a sterile blade and forceps, and placed on water agar to be incubated for 7–14 d at 25 ± 2 °C until the growth of the fungus was visually observed.^22^ Once the fungi were isolated, they were given sample codes and preserved at 4 °C until further use.

### Purification of fungal isolates

Colony morphology of the isolates was observed while sub-culturing. In brief, 1 × 1 cm discs of mycelia grown on water agar plates were excised and inoculated into freshly prepared potato dextrose agar (PDA; HiMedia Laboratories Ltd) plates by the hyphal tip method.^23,24^ Plates were incubated for 7 d at 25 ± 2 °C. Pure isolates with distinct morphological features^25^ were selected for further molecular identification. Pure isolates were preserved in potato dextrose broth (PDB) with glycerol and subcultured in the same media before performing genomic DNA extraction and fermentation experiments. A total of 9 endophytic fungal strains with varying and distinct morphotypes were isolated and screened for catechin production. Among these fungal strains, only two (CSPL6 and CSPL5b) were capable of catechin production and were selected for further studies.

### Molecular identification of isolates

Freshly grown mycelia were harvested and genomic DNA was extracted using the cetyltrimethylammonium bromide (CTAB) method.^26,27^ The internal transcribed spacer (ITS) region of rDNA was amplified using primers specific to ITS1 (5′-TCCGTAGGTGAACCTGCGG-3′) and ITS4 (5′-TCCTCCGCTTATTGATATGC-3′)^28^ and sequenced. Sequence analysis was performed using the BLASTN tool to identify the homologous sequences from the NCBI database. The homologous sequences were aligned using multiple sequence alignment (MSA) and the phylogenetic tree was reconstructed by the neighbor-joining (NJ) method using MEGA 11 software. The bootstrap of 1000 replications was used to analyze the definitive level of the nodes of the tree.

### Fungal culture for secondary metabolite production

The production of secondary metabolites was carried out on a small-scale liquid-state fermentation process using PDB.^29^ Agar plugs (∼5 mm) diameter from a 7 d PDA plate were used as inoculum. Isolates were incubated at 25 ± 2 °C at 100 rpm. After 18–21 d, the mycelium was separated from the fermentation broth using sterile cheesecloth for further extraction procedures.^30^ Secondary metabolites released into the broth by the fungi were extracted into ethyl acetate as per the established method.^31,32^ The dried crude extract was collected into glass vials and stored at 4 °C for further bioassays.

### Targeted chemical characterization of fungal extracts

The total phenolic content was determined using the Folin–Ciocalteu (FC) reagent method using gallic acid as a standard,^33,34^ and the total flavonoid content was measured by aluminum chloride (AlCl_3_) method using quercetin as a standard.^33,34^ The catechin content was measured using HPLC-UV using an established method.^35,36^ In brief, HPLC analysis was performed on a Shimadzu LC-20AD instrument equipped with a DAD detector. Samples were run at 1 mL min^−1^ in a C-18G column (250 × 4.6 mm, 5 μm) in an isocratic mode using a mobile phase of water/acetonitrile/formic acid (899/100/1, v/v, solvent A) and water/acetonitrile/formic acid (699/300/1, v/v, solvent B). The chromatograms were recorded at a wavelength of 280 nm to identify the catechin and EGCG.^37,38^ Quantification was done against the standard curve (*R*^2^ = 0.998) of authentic standards (Sigma).

### Untargeted chemical characterization of fungal extract

Fungal extracts that were found positive for catechin production underwent GCMS-based untargeted chemical fingerprinting following the established method.^39,40^ In brief, dried samples were derivatized using *N*,*O*-bis(trimethylsilyl)trifluoroacetamide + trimethylchlorosilane (99 : 1, v/v) (Sigma) and extracted into ethyl acetate. Samples were run in a Shimadzu QP 2010 Ultra GCMS instrument equipped with a TG-5MS column (30 m × 0.25 mm × 0.25 μm). The injector temperature was set at 250 °C, and the initial temperature of the program was set at 60 °C (solvent delay 5 min) with a hold of 5 min, followed by a ramp of 5 °C to 300 °C with a hold of 5 min. MS data were analyzed using Automated Mass Spectral Deconvolution and Identification System (AMDIS) version 2.70. The major and essential compounds were identified by mass fragmentation patterns (*m*/*z*) of the reference parent compound (molecular peak and base peak) using MS Interpreter version 2.0 and by matching with the reference database of the National Institute of Standards and Technology (NIST) with an MS Library V2011.

### Enrichment and pathway analysis

MetaboAnalyst 5.0 was used to analyze the biochemical pathway enrichment using the metabolite abundance data sets obtained from the GCMS analysis.^41^ Enrichment analysis was performed based on the Kyoto Encyclopedia of Genes and Genomes (KEGG) and was used to investigate how groups of functionally related metabolites are significantly enriched that would potentially eliminate requirements of preselect compounds based on arbitrary cut-off thresholds. Identified metabolites were mapped against PubChem and KEGG identifiers. Pathway analysis was performed based on KEGG identifiers, where out-degree centrality was used for topology analysis, and Fisher's exact test was used as an enrichment method.

### Antioxidant and free-radical scavenging activities

The antioxidant and free-radical scavenging activities of the fungal extracts were evaluated against various physiologically relevant intracellular free-radicals. All assays were performed against appropriate standards as per previously standardized methods. The range of the highest sample dose for each assay was based on the linear response range for respective standard compounds in the final volume of the reaction mixture.

### Scavenging of free-radicals of Haber–Weiss reaction

The Fenton and Haber–Weiss reaction serves as one of the key intracellular free-radical forming reactions generating the highly reactive hydroxyl radical (OH˙), superoxide radical (O_2_˙^−^), and hydrogen peroxide (H_2_O_2_) from oxygen (O_2_) in the presence of transition metal ion (*e.g.*, iron). Therefore, previously standardized *in vitro* assays comprising the formation and scavenging of OH˙, O_2_˙^−^, and H_2_O_2_ were performed.^42,43^ The Fenton reaction of the Fe^3+^–ascorbate–EDTA–H_2_O_2_ system led to the generation of OH˙, which was measured at 532 nm using mannitol as the OH˙ scavenger.^44^ The interaction of phenazine methosulfate (PMS) and reduced nicotinamide adenine dinucleotide (NADH) produces O_2_˙^−^, which was quantified by reducing nitroblue tetrazolium (NBT) to purple-colored formazan. Quercetin was used as a standard.^45^ A FOX reagent test was used to measure the extract's H_2_O_2_ scavenging ability by binding oxidized Fe^2+^ to xylenol orange. The absorbance was measured at 560 nm using sodium pyruvate as standard.^46^ Additionally, an iron chelation assay was performed to evaluate the Fe^2+^ to Fe^3+^ transition potentials of the fungal extracts.^47^ This was important since transition metals (*e.g.*, iron) can accelerate the Fenton reaction by triggering the formation of OH˙ from H_2_O_2_. Iron chelation activity of extracts was assessed by measuring the decreased intensity of the violet complex at 562 nm, resulting from Fe^2+^ and ferrozine coupling.^48^ EDTA was used as standard.

### Scavenging of reactive nitrogen species (RNS)

Nitric oxide (NO) is produced at physiological pH by the reaction between aqueous sodium nitroprusside (SNP) with (O_2_), as measured by the Griess–Ilosvay reaction. A pink azo dye formed was measured at 540 nm with curcumin as the standard.^49^ The peroxynitrite (OONO^−^) scavenging activity of extracts was measured at 611 nm using the Evans blue bleaching assay.^50^ Gallic acid was used as standard.

### Scavenging of reactive oxygen species (ROS)

Hypochlorous acid (HOCl) is produced by reacting NaOCl and H_2_SO_4_ at pH 6.2. The HOCl scavenging activity of extracts was evaluated at 404 nm by monitoring catalase absorbance decrease. Ascorbic acid was used as a standard.^51^ The interaction between NaOCl and H_2_O_2_ produced singlet oxygen (^1^O_2_). The scavenging activity of the fungal extracts was evaluated by monitoring the bleaching of *N*,*N*-dimethyl-4-nitrosoaniline (RNO) at 440 nm and lipoic acid was used as standard.^45^ The overall reducing efficacy of the extracts was measured by monitoring the scavenging of 2,2-diphenyl 1-picrylhydrazyl (DPPH) radical, which serves as a surrogate indicator of the total antioxidant assay of the extracts.^40^ Since intracellular free-radical formation triggers the vicious cycle of membrane lipid peroxidation, the potential of the extracts to prevent OH˙ radical mediate lipid peroxidation was measured using fresh chicken brain samples. All assays were performed in 96-well plates as indicated previously.^40^

### Evaluation of probiotic characteristics of crude fungal extracts

Bacterial strains were procured from the Microbial Type Culture Collection (MTCC) at CSIR-Institute of Microbial Technology, Chandigarh, India. The strains were preserved as frozen (−80 °C) stocks of de Man, Rogosa, and Sharpe (MRS) broth supplemented with 20% (v/v) glycerol. The cultures were successfully transferred three times consecutively using a 1% (v/v) inoculum in MRS broth at 37 °C for 18 h before use. The crude ethyl acetate extracts of the endophytic fungi were tested against probiotic strains *L. plantarum*, *L. reuteri*, *L. rhamnosus*, and *L. sporogenes*. In brief, all of the bacterial suspensions were equivalent to 1.5 × 10^8^ CFU mL^−1^ (0.5 McFarland turbidity standards). A 96-well microplate bioassay was adopted as per a previous study,^52^ to evaluate the prebiotic effects of low (1–8 μg mL^−1^) and high (50 and 100 μg mL^−1^) concentrations of the fungal extracts. Absorbance was recorded at 600 nm in every 2 h interval.

### Statistical analysis

Data were statistically analyzed using GraphPad V8 and quantitative data was represented as mean ± SEM of 3–4 replicates and analyzed by one-way ANOVA otherwise indicated. The inhibitory percentage (%) for the antioxidant assays was calculated by the formula ((*X*_0_ − *X*_1_)/*X*_0_) × 100, where *X*_0_ represents the absorbance of the control and *X*_1_ represents the absorbance of the sample and standard treated wells. The half maximal inhibitory concentration (IC_50_) values were calculated by formula *Y* = *A*_1_/(*X* + *A*_1_) × 100, where *A*_1_ = IC_50_, *Y* = response (*Y* = 100% when *X* = 0), *X* = inhibitory concentration. The metabolite abundance data sets from the GCMS study were utilized to investigate the biochemical pathway enrichment using MetaboAnalyst V5. The observed hit/expected hit values were used to compute the enrichment ratio (ER) for the identified metabolites and pathways. For each enrichment analysis entry, Holm–Bonferroni correction for *P*-value and false discovery rate (FDR) was computed. Fisher's exact test was used to perform the pathway analysis. The enrichment analysis was performed utilizing the KEGG database to explore the significant enrichment of functionally related metabolite classes. The identified metabolites were cross-referenced with identifiers from PubChem. Growth-curve data was analyzed using 2-way repeated measure ANOVA to determine the effects of individual variables of time and concentration and their interaction with time. For every treatment, Tukey's *post hoc* test was applied to compute the differences in area under the curve (AUC_Δ_0–16 h__).

## Results

### Identification of endophytic fungal isolates

**Fig. 2:**
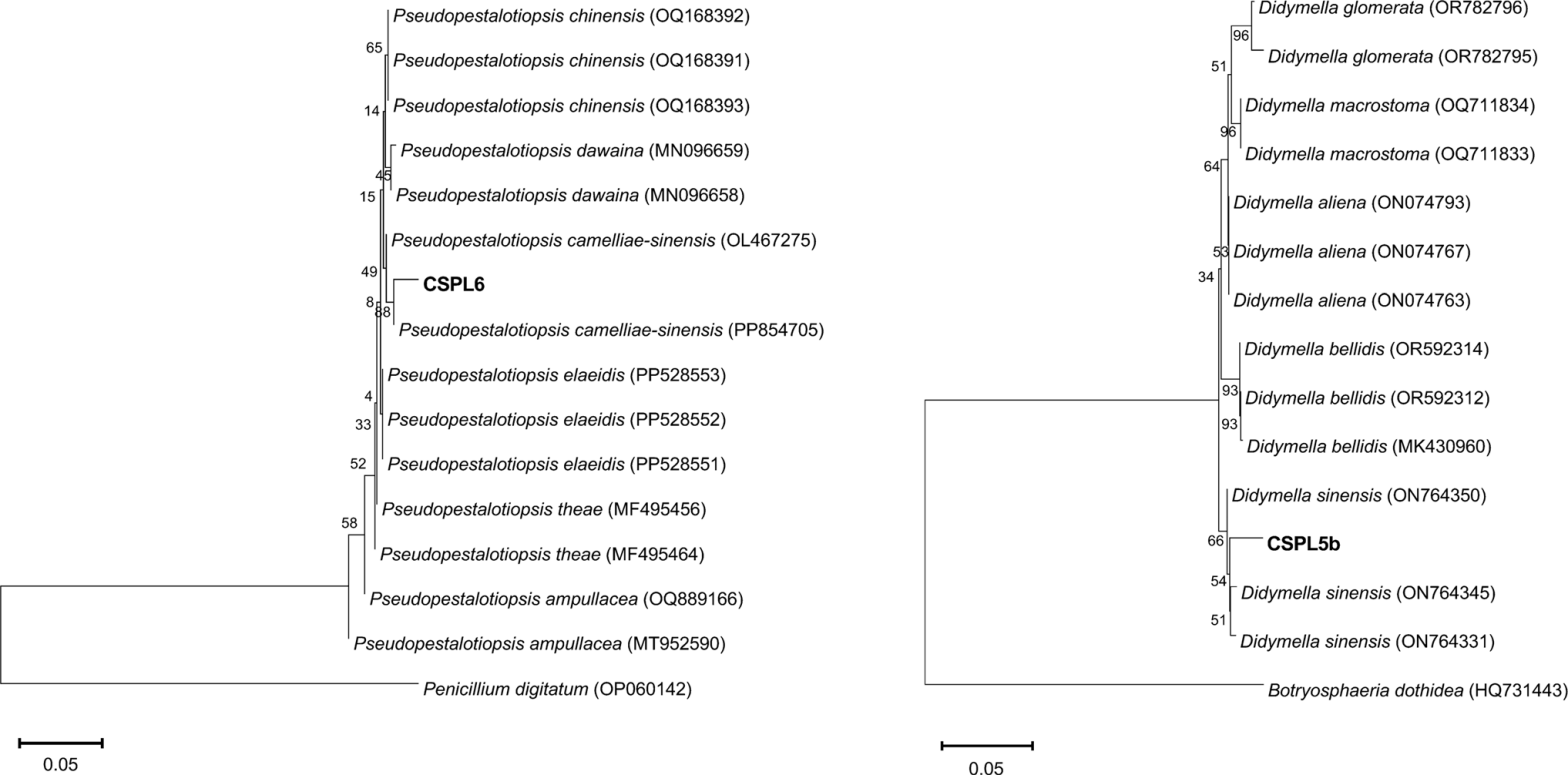
Phylogenetic trees showing the relationships between the ITS sequences of the catechin-producing isolates. Left pannel: CSPL6, and right pannel: CSPL5b (shown in bold), and those of other related fungal species retrieved from GenBank. The tree was constructed based on the rDNA sequence (ITS1 and ITS4) by using the neighbor-joining method.

The two isolates exhibited distinct morphological and microscopic characteristics. CSPL6 displayed a white-colored cotton appearance (Fig. 1C and D). Under microscopic examination, CSPL6 had conidia with uniformly colored cells in shades of brown, dark brown, or olive green (Fig. 1E). CSPL5b exhibited uneven mycelium, with a greenish-brown color towards the middle and a lighter periphery. The fully developed fungus had a uniform brown appearance (Fig. 1F and G). CSPL5b had uniform oval-shaped cells (Fig. 1H). The ITS sequences of the isolates CSPL6 and CSPL5b consisting of 582 and 531 base pairs, were analyzed using BLASTN, which showed similarity (with query coverage) to *Pseudopestalotiopsis camelliae-sinensis* and *Didymella sinensis*, respectively (Fig. 2A and B). Based on the morphological and molecular characteristics, the fungal isolates CSPL6 and CSPL5b were identified as *Pseudopestalotiopsis camelliae-sinensis* and *Didymella sinensis*, respectively. The obtained sequences were deposited in the NCBI database under the accession numbers PP956880 and PP939949, respectively.

### Chemical characterizations

#### Phenolic and flavonoid content

The total phenolic content of the endophytic fungal extracts of CSPL6 and CSPL5b, computed from the calibration curve (*R*^2^ = 0.997), was 21.38 ± 0.11 and 21.01 ± 1.35 μg of gallic acid equivalents per mg of extract, respectively. The flavonoid content of the crude fungal extracts CSPL6 and CSPL5b determined spectrophotometrically from the standard graph (*R*^2^ = 0.998), was 5.60 ± 0.25 and 4.68 ± 0.39 μg of quercetin equivalents per mg of extract, respectively.

#### Estimation of catechins

**Fig. 3:**
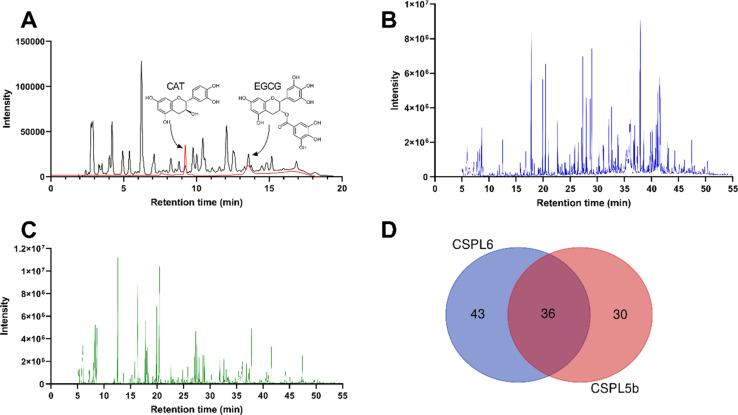
(A) High-performance liquid chromatogram of authenticate catechin and EGCG (in red) and fungal catechin (in black) (B) gas chromatogram of CSPL6 (C) gas chromatogram of CSPL5b (D) Venn diagram demonstrating the number of metabolites identified in each sample and the number of common metabolites.

HPLC profile of the two fungal extracts at 280 nm revealed the presence of catechin and EGCG in the fungal extracts (Fig. 3A). The catechin content in CSPL6 and CSPL5b was calculated to be 381.48 and 166.40 μg mg^−1^ of extract, respectively, whereas EGCG content was calculated to be 484.41 and 281.99 μg mg^−1^ of extract, respectively.

#### Untargeted metabolomics of fungal extracts

A total of 79 and 66 distinct phytochemical metabolites were identified in CSPL6 and CSPL5b, respectively (Fig. 3B, C, ESI Tables 1 and 2†). The top five predominant metabolites were hexadecanoic acid (4.44%), benzeneacetic acid (3.29%), 4-hydroxyphenylethanol (2.84%), butanedioic acid (2.51%), and octadecanoic acid (2.12%) in CSPL6, whereas butanedioic acid (8.15%), propanoic acid (7.5%), butane (5.39%), butanoic acid (4.75%), and benzeneacetic acid (4.54%) were most predominant in CSPL5b. Some of the small phenolic metabolites commonly identified in both extracts include 4-hydroxyphenylethanol, 3-(2-hydroxyethyl)indole, 2-hydroxy-5-nitrobenzyl alcohol, 3-hydroxyanthranilic acid, cinnamic acid, and hydrocinnamic acid. Among other bioactive compounds, 13-*cis*-retinoic acid, camphoric acid, 2-quinolinecarboxylic acid and phloroglucinol in CSPL6, and 1-methyladenosine, benzeneacetic acid and valproic acid were identified in CSPL5b. Azelaic acid and hydrocinnamic acid were commonly found in both extracts. Among the metabolites commonly detected in both samples (Fig. 3D), butanedioic acid, propanoic acid, benzeneacetic acid, hexadecanoic acid, and butanoic acid were the top 5 predominant ones.

#### Enrichment and pathway analysis

**Fig. 4:**
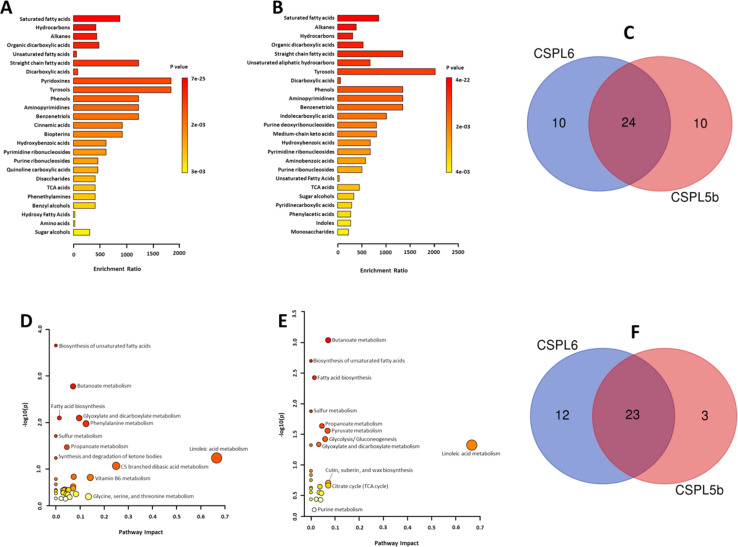
(A) Metabolite enrichment analysis of CSPL6, and (B) CSPL5b at sub-class of chemicals (C) Venn diagram demonstrating the number of sub-classes identified in each extract and the number of common sub-classes identified (D) metabolic pathway enrichment of CSPL6 and (E) CSPL5b (F) Venn diagram demonstrating the number of metabolic pathways identified in each extract and the number of common metabolic pathways identified.

Subjecting the untargeted metabolomics data to bioinformatics tools revealed cumulative enrichment of certain chemical classes in both the extracts against the KEGG database (Fig. 4A, B, ESI Tables 3 and 4†). In both samples, saturated fatty acids, alkanes, and hydrocarbons were the top three significantly enriched metabolites. The methyl derivatives of vitamin B6 (*i.e.*, pyridoxines) were highly enriched in sample CSPL6, but absent in CSPL5b. Similarly, cinnamic acids, biopterins, and quinoline carboxylic acids were enriched in CSPL6, but absent in CSPL5b. Contrarily, unsaturated aliphatic hydrocarbons, indolecarboxylicacids, and medium-chain keto acids were enriched in CSPL5b, but absent in CSPL6. Collectively, 10 uniquely enriched metabolite subclasses were identified in both extracts, while 24 of them were commonly enriched (Fig. 4C).

*In silico* analysis of enriched metabolic pathways revealed linoleic acid metabolism, vitamin B6 metabolism, glycine, serine and threonine metabolism, phenylalanine metabolism, and glyoxylate and dicarboxylate metabolism as the top 5 most enriched pathways in CSPL6 (Fig. 4D and ESI Table 5†). Linoleic acid metabolism, butanoate metabolism, cutin, suberin and wax biosynthesis, citrate cycle (TCA cycle), and pyruvate metabolism were the top five enriched pathways in CSPL5b (Fig. 4E and ESI Table 6†). Although butanoate metabolism, biosynthesis of unsaturated fatty acids (UFAs), and fatty acid biosynthesis in general, were significantly enriched in both extracts, all three pathways differed in their level of enrichment in both extracts. A total of 23 pathways were found common in both extracts (Fig. 4F).

### Antioxidant and free-radical scavenging activities

#### Impact on component free-radicals of Haber–Weiss reaction

**Fig. 5:**
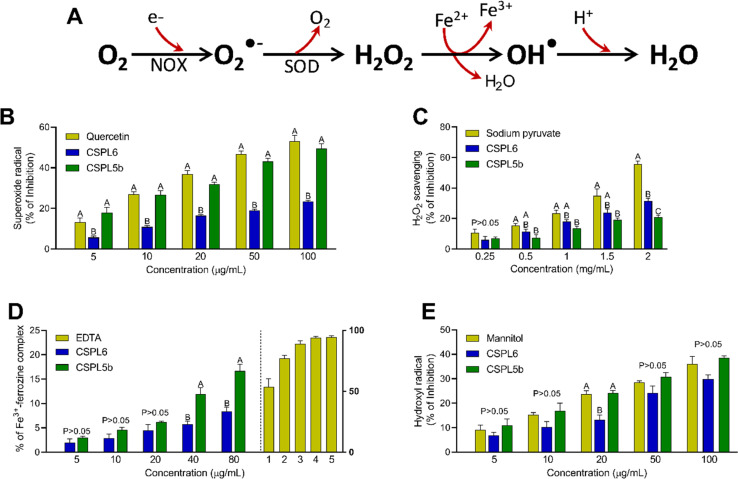
(A) The diagram illustrates how the cells generate free-radicals from oxygen (O_2_). The superoxide radical (O_2_˙^−^), is produced from O_2_ by the mitochondrial NADPH oxidase (NOX). The cytosolic superoxide dismutase enzyme (SOD) converts O_2_˙^−^ into hydrogen peroxide (H_2_O_2_) in the presence of Cu/Zn. When Fe^2+^ is present, H_2_O_2_ undergoes Fenton-reaction, forming highly reactive hydroxyl radical (OH˙) which is then protonated to produce water. Antioxidant and free-radical scavenging activities of (B) superoxide radical scavenging; (C) hydrogen peroxide scavenging; (D) iron chelation; and (E) hydroxyl radical scavenging.

A dose-dependent activity in free-radical scavenging was recorded for both extracts (Fig. 5A–E). For O_2_˙^−^ scavenging, CSPL5b demonstrated comparable effects to the standard quercetin, while CSPL6 showed relatively lower bioactivities as indicated by significantly higher IC_50_ value (Table 1). A similar trend was observed in the case of H_2_O_2_ scavenging, where CSPL5b and standard sodium pyruvate demonstrated comparable IC_50_ that was lower than that of CSPL6. Standard EDTA demonstrated far superior efficacy for iron chelating activity than that of both extracts. While both the extracts demonstrated comparable effects at low concentrations, CSPL5b showed relatively better effects beyond 40 μg mL^−1^. In the case of OH˙ scavenging, although both extracts demonstrated comparable effects to that of mannitol, CSPL5b showed overall superior bioactivity in terms of IC_50_.

**Table 1:** Half maximal inhibitory concentration (IC_50_) of CSPL6, CSPL5b, and standards for different antioxidant and free radical scavenging assays. Data expressed as mean ± S.E. (*n* = 3). *p < 0.05; ns *vs.* standard. Data expressed as mean ± S.E. (*n* = 3). A = high IC_50_; B = low IC_50_ value [within extracts]

Parameters	Extracts^a^		Standard
	CSPL6	CSPL5b	
DPPH	140.1 ± 3.98***^A^	98.54 ± 1.42***^B^	39.66 ± 1.33 (ascorbic acid)
Hydroxyl (OH˙)	163.62 ± 11.57^nsA^	124.10 ± 6.32^nsB^	135.92 ± 13.22 (mannitol)
Superoxide (O_2_˙^−^)	221.58 ± 4.57***^A^	86.40 ± 2.85^nsB^	77.36 ± 4.72 (quercetin)
Hydrogen peroxide (H_2_O_2_)	3.22 ± 0.22**^B^	4.69 ± 0.19***^A^	1.93 ± 0.03 (sodium pyruvate)
Nitric oxide (NO)	101.87 ± 4.94**^A^	73.98 ± 3.39^nsB^	58.88 ± 5.83 (curcumin)
Peroxynitrite (OONO^−^)	322.87 ± 55.40^nsB^	515.96 ± 99.42^nsA^	329.39 ± 46.38 (gallic acid)
Singlet oxygen (^1^O_2_)	471 ± 24.04***^A^	223.30 ± 12.57***^B^	96.68 ± 3.37 (lipoic acid)
Hypochlorous acid (HOCl)	181.41 ± 34.93*^A^	96.76 ± 6.45*^B^	75.45 ± 3.45 (ascorbic acid)
Lipid peroxidation	219.76 ± 30.64**^A^	58.56 ± 3.77**^B^	34.18 ± 1.37 (trolox)
Ferrous chelation	532.27 ± 53.73***^A^	245.10 ± 21.34***^B^	1.45 ± 0.11 (EDTA)

a All units in μg mL^−1^ except for H_2_O_2_ scavenging (mg mL^−1^).

#### Impact on reactive nitrogen species

**Fig. 6:**
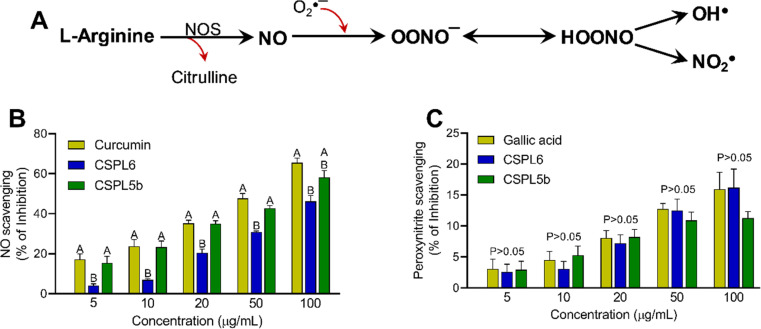
(A) The diagram depicts that the enzyme nitric oxide synthase (NOS) produces nitric oxide (NO) from l-arginine in the presence of O_2_. O_2_˙^−^ and NO react to produce peroxynitrite anion (OONO^−^). Additionally, peroxynitrous acid (HOONO) breaks into OH˙ and NO_2_˙. Antioxidant and free-radical scavenging activities of (B) nitric oxide scavenging and (C) peroxynitrite scavenging.

CSPL5b demonstrated NO scavenging effects comparable to standard curcumin and was statistically superior to CSPL6 across most of the tested concentrations (Fig. 6A–C). This was also reflected by the comparable IC_50_ of CSPL5b and curcumin which was significantly lower than that of CSPL6 (Table 1). Contrarily, in the case of OONO^−^ scavenging, despite the dose-dependent effect, although no statistical difference was observed between the extracts and standard gallic acid across all concentrations, CSPL6 had better IC_50_ compared to CSPL5b.

#### Other antioxidant effects

**Fig. 7:**
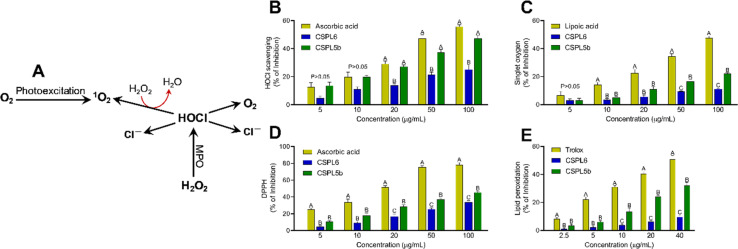
(A) The flowchart depicts the production of singlet oxygen (^1^O_2_) from O_2_ by photoexcitation method. Alternatively, H_2_O_2_ can undergo a reaction with myeloperoxidase (MPO) to produce hypochlorous acid (HOCl) which then decomposes into chloride ions (Cl^−^) and ^1^O_2_. Antioxidant and free-radical scavenging activities of (B) hypochlorous acid scavenging; (C) singlet oxygen scavenging; (D) DPPH assay; and (E) lipid peroxidation assay.

In the case of HOCl scavenging, both extracts showed statistically comparable effects to the standard ascorbic acid at a low dose (Fig. 7A and B). However, beyond 20 μg mL^−1^, CSPL5b demonstrated relatively higher bioactivity than CSPL6, which is also reflected through the lower IC_50_ of CSPL5b (Table 1). Compared to CSPL6, CSPL5b was superior in terms of ^1^O_2_ scavenging (Fig. 7C). However, standard lipoic acid demonstrated better efficacy compared to both extracts. CSPL5b demonstrated comparatively superior reducing power than CSPL6 in terms of scavenging DPPH radical (Fig. 7D) which is also reflected by the statistically lower IC_50_ of CSPL5b (Table 1). Finally, CSPL5b showed statistically better inhibition for OH˙ induced peroxidation of tissue lipid than CSPL6 (Fig. 7E). Nevertheless, standard trolox better-inhibited lipid peroxidation relative to both the extracts.

### Prebiotic effects

**Fig. 8:**
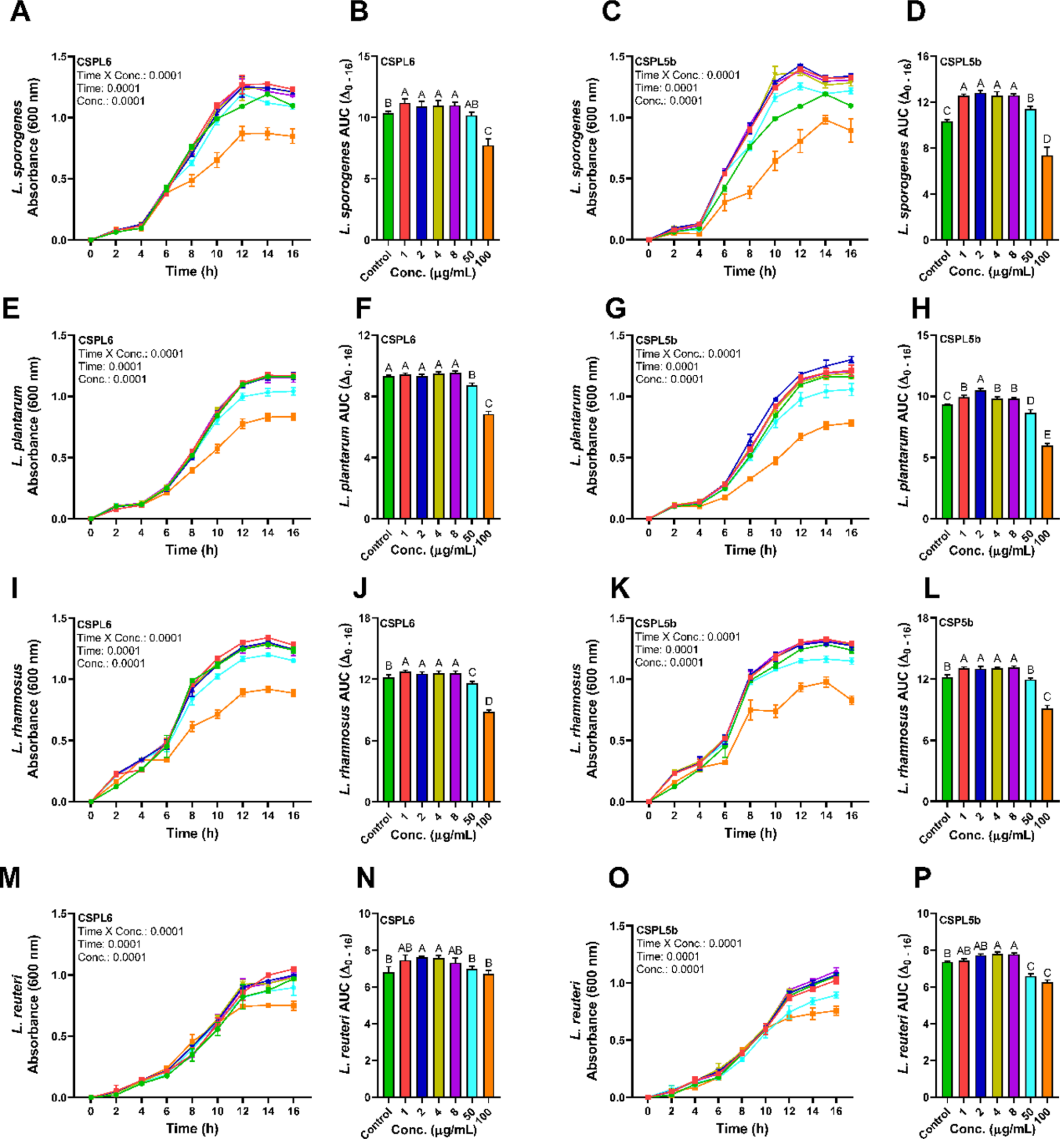
Growth curves and corresponding AUCs of (A and B) *L. sporogenes* grown under CSPL6 and (C and D) CSPL5b fungal extract treatment at different concentrations; (E and F) *L. plantarum* grown under CSPL6 and (G and H) CSPL5b fungal extract treatment at different concentrations; (I and J) *L. rhamnosus* grown under CSPL6 and (K and L) CSPL5b fungal extract treatment at different concentrations; (M and N) *L. reuteri* grown under CSPL6 and (O and P) CSPL5b fungal extract treatment at different concentrations. The data was evaluated using 2-way repeated measure ANOVA to determine the effects of individual variables and their interaction with time. For every treatment, Tukey's *post hoc* test was applied to compute the differences in AUC_(Δ_0–16 h_)_.

The extracts of the catechin-producing endophytes demonstrated significant dose and time-dependent effects on the growth of the probiotic *Lactobacillus* spp. (Fig. 8A–P). Except for CSPL6 on *L. plantarum* growth, compared to control, all extracts demonstrated prebiotic effects at low dose (1–8 μg mL^−1^), while the growth of the probiotic strains was suppressed under high dose (100 μg mL^−1^) except for CSPL6 for *L. reuteri*. CSPL5b at 2 μg mL^−1^ demonstrated the highest impact on the growth of *L. sporogenes* with a 1.28 times increase, followed by 1.15 times higher growth for *L. plantarum* at the same dose. In fact, at 50 μg mL^−1^, CSPL5b demonstrated higher growth enrichment for *L. sporogenes* relative to control. However, at 100 μg mL^−1^, all treatments significantly reduced bacterial growth except for CSPL6 for *L. reuteri*. The prebiotic effects were evident only at low concentrations (1–8 μg mL^−1^), while a growth inhibitory effect was observed under relatively higher concentrations (50 and 100 μg mL^−1^). For all treatments, both CSPL6 and CSPL5b demonstrated a significant (*P* < 0.0001) time and concentration-dependent impact on the growth curve of the probiotic bacteria. Compared to untreated control, the cumulative AUC_(Δ_0–16 h_)_ of the growth curve shows significantly higher AUC for the majority of the treatments.

## Discussions

Tea-derived endophytes mostly belonging to the genera *Chryseobacterium*, *Sphingomonas*, *Rhizobium*, *Morganella*, *Methylobacterium*, Comamonadaceae, *Colletotrichum*, *Uwebraunia*, *Cladosporium*, and *Devriesia*,^53^ albeit the majority of which have never been screened for catechin production or bioactivities. Some of the previously isolated endophytic fungi from *C. sinensis* include *Colletotrichum gloeosporioides*,^27^*Cladosporium*, *Zymoseptoria*, and *Strelitziana*,^54^ and *Phyllosticta capitalensis*, *Colletotrichum camelliae*, and a *Pleosporales* spp.^55^ Nevertheless, the diversity of tea endophytic fungi is known to be low, which is likely attributed to the commercial tea plantation practices that utilize agrochemicals, impacting the fungal population.^13^ Isolation of endophytes from organically cultivated plants provides an opportunity to negate the adaptation effects of endophytes under the influence of agrochemicals and reflect the effects of inter-kingdom horizontal gene transfers.^56^ Indeed, for the current study, this was important since endophytes acquire phytochemical biosynthesizing genes from the parent plant primarily through horizontal gene transfer.^1^*Pseudopestalotiopsis* and *Didymella* spp. have previously been identified in tea plants,^57–59^ but detailed metabolomics characterization, including the potential to produce catechins, has never been investigated before. Both the endophyte varieties have been, however, attributed as tea-exclusive pathogens causing blight disease to *C. sinensis*.^58,60^ This is relevant since plant biosynthesis of secondary metabolites is in part, associated with the host defense system. Indeed, activation of specific transcription factors regulating catechin biosynthesis has been related to the pathogenic stress in *C. sinensis*.^61^

Contrarily, an array of endophytic fungi, including that of *Penicillium*, *Aspergillus*, and *Fusarium* spp., isolated from *C. sinensis* have been demonstrated with plant growth-promoting potentials through mechanisms involving production of growth promoters,^14^ while *Pseudocercospora kaki* and *Penicillium sclerotiorum* isolated from tea leaves were able to inhibit blast disease-causing pathogen *Magnaporthe grisea*.^62^ Others have reported that endophytic *Penicillium* spp. isolated from tea plants possess phosphate solubilization, siderophore production, and extracellular enzyme-producing properties, that could be harnessed for industrial utility.^63^ Despite these potentials, detailed metabolomic fingerprinting of the tea-derived endophytic fungi, including the study of biochemical pathways and their bioactivities remained underexplored.

In the present study, *P. camelliae-sinensis* and *D. sinensis* were found to produce significant amounts of catechin and EGCG, which were comparable to that of *C. sinensis*.^5^ Reports of alternative sources of catechin are rare except for the plants belonging to the genus *Acacia*.^64^ Although for the first time, we report the presence of catechin-producing endophytic fungi isolated from tea leaves, to our knowledge, there have been only two reports on catechin-producing endophytes, *Annulohypoxylon elevatidiscus*, and *A. ilanense*, isolated from decorticated woods collection of unknown floral origin.^16,17^ Nevertheless, reports of diverse pharmacologically relevant secondary metabolite-producing endophytes isolated from tea are available. For instance, *Alternaria alternata* isolated from tea leaves were shown to produce altenuene and isocoumarin derivatives and were able to inhibit the growth of several pathogenic microbes and demonstrated cytotoxic effects against human osteosarcoma tumor cells and hepatocellular carcinoma cell lines.^65^ Additionally, reports of flavonoid-producing endophytes and associated bioactivities from alternate sources have been reported. For instance, 3-methoxyflavone, nobiletin, formononetin, scopoletin, and daidzein-forming 21 varieties of endophytes were isolated from *Conyza blinii*, having potent antioxidant and antibacterial activities.^66^ Endophyte *Chaetomium globosum* isolated from *Moringa oleifera* demonstrated anti-mutagenicity, cytotoxicity, and antioxidant activities.^67^

Not only that endophytes through endogenous metabolites impact the growth of tea plants by directly impacting energy metabolism and stress tolerance but also acquire genes associated with secondary metabolite biosynthesis from the host plant,^68,69^ we intended to understand the secondary metabolite profile of the isolated endophytes using an untargeted technique. This is supported by a prior study demonstrating tea endophyte *Luteibacter* spp. enhancing the biosynthesis of theanine, the main flavoring compound in tea.^70^ Moreover, in recent times, endophyte-centered metabolic engineering strategies have been employed to enhance the production of pharmacologically relevant secondary metabolites.^71^ We identified an array of bioactive phytochemicals produced by the endophytes which were likely triggered by the specifically enriched metabolic pathways. For instance, the enrichment of C18 (UFAs) biosynthetic pathways, associated with the production of pharmacologically important metabolites, observed in both the fungi, is relevant since they are known to serve as intrinsic antioxidants and precursors of bioactive phytochemicals.^40,72^ Indeed, metabolic enrichment of fatty acid biosynthetic pathway has been proposed for improving the commercial value of plants^73^ and increased sequestration of bioactive metabolites due to enrichment of fatty acid biosynthesis has been reported in several cases.^74,75^ UFAs can also directly scavenge free-radicals and oxylipins, the breakdown products of UFA, that in turn can serve as intrinsic regulators of oxidative stress.^76^ Additionally, we observed the enrichment of butanoate metabolism in both the isolates. This is relevant since it is strongly associated with flavonoid biosynthesis and stress tolerance.^77,78^ Furthermore, both isolates demonstrated enrichment of linoleic acid metabolism which is associated with increased antioxidant effects and stress tolerance through cytoprotective activities.^79,80^ Interestingly, glyoxylate and dicarboxylate metabolism were enriched in both isolates which was otherwise reported to be enriched in *C. sinensis* under N_2_ deficiency.^81^ In line with the identification of various bioactive compounds in both the isolates, the enrichment of glyoxylate and dicarboxylate metabolism is relevant since earlier data suggest increased accumulation of bioactive compounds (*e.g.*, flavonoids) under N_2_ deficient conditions,^82^ likely leading to improved antioxidant and cytoprotective effects.^83^ Enrichment of such crucial biosynthetic and metabolic pathways in the fungal isolates not only ensures possibilities for enhancement of bioactive secondary metabolite production but also indicates that close association of these endophytes with the tea plant likely enhances the stress tolerance of host plant and influences the overall bioactivities of the tea extracts.^84^

Next, we intended to evaluate the antioxidant and free-radical scavenging activities of the tea-derived catechin-producing endophytes since the bioactivities of tea are centered around antioxidant effects, attributed to the presence of catechins. First, we focused on the components free-radicals Haber–Wess reaction since it represents the nucleus of highly reactive O_2_˙^−^ and OH˙ formation. Data showed that CSPL5b demonstrated O_2_˙^−^ scavenging activity comparable to that of standard, while in the case of OH˙, both CSPL6 and CSPL5b showed superior bioactivities. This is important since earlier data suggested that the antioxidant activities of dietary phytometabolites, especially flavonoids, are dictated by the O_2_˙^−^ scavenging activity^75^ and that efficient scavenging of OH˙ could be protective against intracellular lipid peroxidation which is primarily attributed to the highly reactive OH˙.^85^ In aerobes, ^1^O_2_ is produced through the rapid uptake of molecular O_2_ by NADPH oxidase (NOX) through the process of mitochondrial respiration, followed by conversion to H_2_O_2_ by superoxide dismutase (SOD).^86^ The significance of O_2_˙^−^ scavenging is evident from the direct correlation between the lack of cytosolic SOD and chronic inflammatory diseases.

CSPL5b demonstrated better H_2_O_2_ scavenging effects relative to CSPL6. H_2_O_2_ is known to oxidize the thiol groups of intracellular proteins and increased intracellular concentration of H_2_O_2_ under cytoprotection-compromised conditions can lead to the overproduction of OH˙.^87^ CSPL6 and CSPL5b-mediated direct scavenging of H_2_O_2_ therefore likely result in reduced oxidative damage to the cellular biomolecules. Our data also showed that both extracts were able to chelate ionic iron in a dose-dependent manner, however, CSPL5b demonstrated better efficacy than CSPL6. Since transition metals like iron play a major role as a catalyst to promote OH˙ formation, increased Fe^3+^ would be helpful to limit the progress of the Fenton reaction.

Based on IC_50_ values, apart from OONO^−^ scavenging, CSPL5b demonstrated superior bioactivities in the remaining antioxidant assays. NO plays an important role as a pro-inflammatory mediator and can react with intracellular O_2_˙^−^ to generate highly reactive OONO^−^. Although, CSPL6 showed superior effects in the scavenging of OONO^−^*in vitro*, however under intracellular conditions CSPL5b is expected to demonstrate better efficacy in neutralizing OONO^−^ since it demonstrated better efficacy in scavenging NO and O_2_˙^−^ as the precursors to OONO^−^. Data also showed that compared to CSPL6, CSPL5b was superior in scavenging both HOCl and ^1^O_2_. Neutrophils and activated M1 macrophages, during inflammation, produce HOCl through the myeloperoxidase reaction as part of a respiratory burst, causing localized tissue damage.^88^^1^O_2_ on the other hand, has dual origin *viz.* HOCl breakdown and photoexcitation of molecular O_2_ can readily damage cellular biomolecules due to factors such as relatively longer half-life, free transition across non-polar microenvironments, and strong electrophonic nature.^89^ Finally, the superior antioxidant and free-radical scavenging effects of CSPL5b over CSPL6 were demonstrated by the fact that CSPL5b had comparatively better reducing power in the DPPH assay, and was able to prevent lipid peroxidation compared to CSPL6. It is noteworthy that beyond the catechins, several other bioactive phytochemicals were identified in the fungal extracts which synergistically and additively likely contributed to the observed antioxidant effects. Although a detailed discussion on the bioactivities of the phytochemicals is out of the scope of the current manuscript, reports of evidence-based pharmacological effects of the phytochemicals have been summarized in ESI Table S7.†

Based on the previous reports on the prebiotic and antimicrobial effects of catechins, especially on the gut microbiota,^35,90^ we intended to test the effects of the catechin-rich fungal extracts on the growth parameters of the beneficial *Lactobacillus* spp. This was also relevant since the catechin-producing endophytes were specifically isolated from the tea leaves, which are known to exert metabolic health benefits primarily through catechin-mediated intestinal-level effects.^5^ We monitored bacterial growth till 16 h based on the fact that gastrointestinal (GI) retention of drugs and nutrients at the proximal intestine is of comparable duration^91^ and that the mammalian proximal intestine harbors the greatest concentration of *Lactobacillus* spp.^92^ Data showed that, in general, both extracts demonstrated prebiotic effects at low doses (1–8 μg mL^−1^), while bacterial growth retardation was observed under high doses only (100 μg mL^−1^). Specifically, CSPL5b significantly enriched the growth of *L. sporogenes* and *L. plantarum*. These prebiotic effects were further supported by earlier data demonstrating that phytoextract rich in catechin enhances the growth of gut commensals, especially the populations of prebiotics like *Bifidobacterium*, *Lactobacillus*, and *Akkermansia*.^36^ Recent studies show that synbiotic formulations containing *Lactobacillus* spp. and EGCG could reduce inflammation, confer cytoprotective antioxidative effects, and exert hypolipidemic effects.^93,94^ Nevertheless, the EGCG-rich extract at high doses could be detrimental for the probiotic strains as shown in previous studies of antibacterial effects of EGCG at relatively higher doses, including on that of probiotic spp.^93,95^ Our data demonstrating the prebiotic effects of EGCG-rich extract at lower concentrations is relevant since the gastrointestinal concentration of EGCG at the physiological level is low due to rapid phase I and phase II metabolism, and gut microbiota-dependent breakdown of EGCG into smaller phenolics.^5^ Therefore, the prebiotic effects demonstrated by the EGCG-rich extract could be beneficial for the enrichment of gut commensals, especially the beneficial probiotic spp.

## Conclusion

In the present study, catechin and EGCG-producing two endophytic fungi were isolated from organically-grown tea leaves, followed by detailed chemical characterization and assessed their biological activities. Untargeted metabolomics identified the presence of various pharmacologically relevant metabolites, based on which the putative metabolic pathway regulation was identified. The fungal extracts had strong antioxidant activities, efficiently eliminating significant intracellular free-radicals that are relevant to physiological processes, and showing beneficial effects on gut microorganisms as prebiotics. Collectively, this study emphasized the significance of obtaining endophytes from organically grown plants to counteract the effects of agrochemicals on fungal diversity. The isolated endophytes remarkably produce catechins, which are the main bioactive component of tea but have never been reported before in tea-derived endophytes. Thus, alternative to *C. sinensis*, the isolated endophytes could be used as an alternative source of bioactive catechins, while metabolic and genetic engineering strategies could be employed to enhance the catechin production in the endophytes for future pharmacological use.

## Data availability

Raw data of GCMS is available at https://rb.gy/e3q1b5. Remaining relevant raw data are provided in the ESI.†

## Author contributions

MV and PD conceptualized, designed, and supervised the study. DS conducted the investigation, data curation, formal analysis and wrote the first draft of the manuscript. MV and PD edited the final version of the manuscript. All authors have read and agreed to publish the current version of the manuscript.

## Conflicts of interest

The authors declare that there are no conflicts of interest.

## Supplementary Material

RA-014-D4RA05758A-s001
