# Supplementary material for: Chemical characterization, pathway enrichments and bioactive potentials of catechin-producing endophytic fungi isolated from tea leaves
Source: RSC Adv. 2024 Oct 21;14(45):33034–47. doi: 10.1039/d4ra05758a (PMC11492194; doi:10.1039/d4ra05758a)
Supplement: RA-014-D4RA05758A-s001 [file RA-014-D4RA05758A-s001.pdf]

Supplementary Information (SI) for RSC Advances.
This journal is © The Royal Society of Chemistry 2024

Chemical characterization, pathway enrichments and bioactive potentials of catechin-producing endophytic fungi isolated from tea leaves

Dwinder Sidhu[1], M. Vasundhara[1,*], Priyankar Dey[1,*]

[1] Department of Biotechnology, Thapar Institute of Engineering & Technology, Patiala, India-147004

*Corresponding Authors
Priyankar Dey, Ph.D.
M. Vasundhara, Ph.D.
Department of Biotechnology,
Thapar Institute of Engineering & Technology,
Patiala, Punjab, India
Email: priyankar.dey@thapar.edu; mvasundhara@thapar.edu
Mob: +91-9064275660; +91-8146480908

Supplementary data

Table S1. Compounds identified in CSPL6. The corresponding chromatogram is in Fig. 3B.

S.No.	Name of Compound	Retention time (min)	Relative area (%)	Chemical formula	M.W.
1	Propanoate	5.28	0.14	$C_3H_6O_2$	74.08
2	Butanoic acid	8.63	1.12	$C_4H_8O_2$	88.11
3	Butane	11.92	0.23	C_4H_{10}	58.12
4	Decane	12.42	0.07	$C_{10}H_{22}$	142.28
5	Propanoic acid	12.53	0.75	$C_3H_6O_2$	74.08
6	Undecane	13.88	0.09	$C_{11}H_{24}$	156.31
7	2-Methyl-pyridine	16.50	0.10	C_6H_7N	93.13
8	Pantoyl lactone	16.52	0.06	$C_6H_{10}O_3$	130.14
9	Dodecane	17.05	0.15	$C_{12}H_{26}$	170.33
10	Pentanoic acid	18.06	0.32	$C_5H_{10}O_2$	102.13
11	Glycerol	19.22	0.15	$C_3H_8O_3$	92.09
12	2-methylpyran-4-one	19.52	0.16	$C_6H_6O_2$	110.11
13	Butanedioic acid	20.41	2.51	$C_4H_6O_4$	118.09
14	Pentadecane	20.65	0.09	$C_{15}H_{32}$	212.41
15	Benzaldehyde	22.09	0.07	C_7H_{6O}	106.12
16	1-Dodecene	22.67	1.33	$C_{12}H_{24}$	168.32
17	Tetradecane	22.87	0.25	$C_{14}H_{30}$	198.39
18	Pentanedioic acid	22.94	0.07	$C_5H_8O_4$	132.11
19	Methylmaleic acid	23.43	0.19	$C_5H_6O_4$	130.10
20	3-ketobutyrate	23.98	0.24	$C_4H_6O_3$	102.09
21	Octanoic acid	24.17	0.11	$C_8H_{16}O_2$	144.21
22	Phenol	25.80	0.83	C_6H_6O	94.11
23	L-Proline	25.98	0.11	$C_5H_9NO_2$	115.13
24	4-Pyrimidinamine	26.40	0.08	$C_4H_5N_3$	95.10
25	Acetic acid	27.12	1.07	$C_2H_4O_2$	60.05
26	1-Dodecanol	27.26	0.26	$C_{12}H_{26}O$	186.33
27	4-Hydroxyphenylethanol	27.30	2.84	$C_8H_{10}O_2$	138.16
28	Hexadecen-1-ol	27.91	1.71	$C_{16}H_{32}O$	240.42
29	Phloroglucinol	28.05	0.16	$C_6H_6O_3$	126.11
30	Hexadecane	28.06	0.29	$C_{16}H_{34}$	226.44
31	Heptadecane	28.07	0.27	$C_{17}H_{36}$	240.47
32	Benzoic acid	28.68	1.61	$C_7H_6O_2$	122.12
33	Benzeneacetic acid	28.98	3.30	$C_8H_8O_2$	136.15
34	Dodecanoic acid	29.27	0.08	$C_{12}H_{24}O_2$	200.32
35	L-Threonic acid	29.91	0.11	$C_4H_8O_5$	136.10
36	11,14-Eicosadienoic acid	30.45	0.12	$C_{20}H_{36}O_2$	308.50
37	Phosphorin	30.76	0.05	C_5H_5P	96.07
38	Glutaconic acid	31.06	0.23	$C_5H_6O_4$	130.10
39	Nonanedioic acid	31.31	0.05	$C_9H_{16}O_4$	188.22

40	Hydrocinnamic acid	31.75	0.12	$C_9H_{10}O_2$	150.17
41	Azelaic acid	32.59	0.11	$C_9H_{16}O_4$	188.22
42	1-Nonadecene	32.60	1.27	$C_{19}H_{38}$	266.50
43	Carbamic acid	33.89	0.05	CH_3NO_2	61.04
44	D-Mannopyranose	33.97	0.08	$C_6H_{12}O_6$	180.16
45	13-cis-Retinoic acid	34.42	0.21	$C_{20}H_{28}O_2$	300.44
46	α-D-Glucopyranoside	34.88	0.13	$C_6H_{11}O_6^-$	179.15
47	3-Hydroxyanthranilic acid	34.93	0.07	$C_7H_7NO_3$	153.14
48	Methylglutaconic acid	35.27	0.26	$C_6H_8O_4$	144.13
49	Cinnamic acid	35.67	0.12	$C_9H_8O_2$	148.16
50	n-Pentadecanoic acid	35.84	0.06	$C_{15}H_{30}O_2$	242.40
51	2-Undecenoic acid	35.94	0.32	$C_{11}H_{20}O_2$	184.28
52	10-Undecenoic acid	35.94	0.27	$C_{11}H_{20}O_2$	184.28
53	D-(+)-trehalose	35.967	0.06	$C_{12}H_{22}O_{11}$	342.30
54	2-Quinolinecarboxylic acid	36.71	0.50	$C_{10}H_7NO_2$	173.17
55	Heneicosane	36.98	0.15	$C_{21}H_{44}$	296.57
56	Palmitelaidic acid	37.41	0.50	$C_{16}H_{30}O_2$	254.41
57	Hexadecanoic acid	37.91	4.44	$C_{16}H_{32}O_2$	256.42
58	Trinexapac-ethyl	38.67	0.06	$C_{13}H_{16}O_5$	252.26
59	2-Hydroxy-5-nitrobenzyl alcohol	39.42	0.18	$C_7H_7NO_4$	169.13
60	D-Galactopyranose	39.73	0.29	$C_6H_{12}O_6$	180.16
61	1,2,4-Triazole	40.05	0.06	$C_2H_3N_3$	69.07
62	1-Docosene	40.74	0.47	$C_{22}H_{44}$	308.59
63	Glycine	40.87	0.91	$C_2H_5NO_2$	75.07
64	9,12-Octadecadienoic acid	40.96	0.09	$C_{18}H_{32}O_2$	280.40
65	11-cis-Octadecenoic acid	41.21	0.11	$C_{18}H_{34}O_2$	282.50
66	3-methylvaleric acid	41.27	0.08	$C_6H_{12}O_2$	116.16
67	Camphoric acid	41.32	0.13	$C_{10}H_{16}O_4$	200.23
68	Octadecanoic acid	41.63	2.12	$C_{18}H_{36}O_2$	284.48
69	Tetratriacontane	41.66	0.08	$C_{34}H_{70}$	478.92
70	2,5-Piperazinedione	42.13	0.05	$C_4H_6N_2O_2$	114.10
71	Tetradecanoic acid	42.98	0.78	$C_{14}H_{28}O_2$	228.37
72	Octacosanol	44.29	0.29	$C_{28}H_{58}O$	410.76
73	Uridine	45.13	0.18	$C_9H_{12}N_2O_6$	244.20
74	Linolenic acid	45.68	0.07	$C_{18}H_{30}O_2$	278.43
75	5α-Androstan-17-one	45.95	0.09	$C_{19}H_{30}O$	274.40
76	1-Methyladenosine	47.44	0.78	$C_{11}H_{15}N_5O_4$	281.27
77	Pregn-4-ene-3,20-dione	47.94	0.14	$C_{21}H_{30}O_2$	316.46
78	2-Amino-1-phenylethanol	49.79	0.12	$C_8H_{11}NO$	137.18
79	Benzeneethanamine	49.79	0.16	$C_8H_{11}N$	121.18

21

Table S2. Compounds identified in CSPL5b. The corresponding chromatogram is in Fig. 3C.

S.No.	Compound Name	Retention time (min)	Relative area (%)	Molecular formula	M.W.
1	2-Propenoic acid	5.17	0.29	$C_3H_4O_2$	72.06
2	Propanoate	5.35	0.83	$C_3H_6O_2$	74.08
3	L-(+)-Lactic acid	7.64	0.07	$C_3H_6O_3$	90.08
4	2,3-Butanediol	7.83	0.11	$C_4H_{10}O_2$	90.12
5	Butanoic acid	8.33	4.75	$C_4H_8O_2$	88.11
6	Propanoic acid	12.62	7.50	$C_3H_6O_2$	74.08
7	Butane	16.38	5.40	C_4H_{10}	58.12
8	n-octene	16.46	0.18	C_8H_{16}	112.21
9	1-Dodecene	16.80	0.26	$C_{12}H_{24}$	168.32
10	Dodecane	17.05	0.07	$C_{12}H_{26}$	170.33
11	Pentanoic acid	18.03	1.42	$C_5H_{10}O_2$	102.13
12	5-Nonanol	18.09	0.28	$C_9H_{20}O$	144.25
13	Ethyl succinate	18.45	0.05	$C_6H_8O_4^{-2}$	144.12
14	Glycerol	19.24	0.31	$C_3H_8O_3$	92.09
15	3-Pyridinecarboxylic acid	19.79	0.32	$C_6H_5NO_2$	123.11
16	1,3-Dioxolane	19.92	0.14	$C_3H_6O_2$	74.08
17	Benzeneacetic acid	19.96	4.54	$C_8H_8O_2$	136.15
18	Valproic acid	20.27	0.34	$C_8H_{16}O_2$	144.21
19	Butanedioic acid	20.51	8.15	$C_4H_6O_4$	118.09
20	Benzaldehyde	22.10	0.13	C_7H_6O	106.12
21	1-Tridecene	22.67	0.91	$C_{13}H_{26}$	182.35
22	Tetradecane	22.87	0.13	$C_{14}H_{30}$	198.39
23	Pentanedioic acid	22.95	0.16	$C_5H_8O_4$	132.11
24	D-Isoleucine	23.83	0.15	$C_6H_{13}NO_2$	131.17
25	3-ketobutyrate	23.99	0.23	$C_4H_6O_3$	102.09
26	4H-Pyran-4-one	24.89	0.60	$C_5H_4O_2$	96.08
27	Gly-leu	25.11	0.08	$C_8H_{16}N_2O_3$	188.22
28	Phenol	25.81	0.87	C_6H_6O	94.11
29	4-Pyrimidinamine	26.41	0.05	$C_4H_5N_3$	95.10
30	Acetic acid	27.13	1.37	$C_2H_4O_2$	60.05
31	1-Dodecanol	27.26	0.23	$C_{12}H_{26}O$	186.33
32	4-Hydroxyphenylethanol	27.30	2.59	$C_8H_{10}O_2$	138.16
33	1-Hexadecene	27.92	1.23	$C_{16}H_{32}$	224.42
34	Hexadecane	28.07	0.15	$C_{16}H_{34}$	226.44
35	6-Amino-1-hexanol	28.33	0.14	$C_6H_{15}NO$	117.19
36	Heptanedioic acid	28.43	0.05	$C_7H_{12}O_4$	160.17
37	Benzoic acid	28.68	1.30	$C_7H_6O_2$	122.12
38	Dodecanoic acid	29.27	0.07	$C_{12}H_{24}O_2$	200.32
39	Ala-Thr	29.43	0.10	$C_7H_{14}N_2O_4$	190.20
40	Octanedioic acid	30.33	0.11	$C_8H_{14}O_4$	174.19
41	Azelaic acid	32.58	0.24	$C_9H_{16}O_4$	188.22
42	1-Nonadecene	32.61	1.23	$C_{19}H_{38}$	266.50
43	Octadecane	32.74	0.13	$C_{18}H_{38}$	254.49

44	N-Acetyl-L-phenylalanine	32.85	0.09	$C_{11}H_{13}NO_3$	207.23
45	3-(2-Hydroxyethyl)indole	33.45	0.34	$C_{10}H_{11}NO$	161.20
46	Tetradecanoic acid	33.74	0.17	$C_{14}H_{28}O_2$	228.37
47	Hydrocinnamic acid	34.25	0.11	$C_9H_{10}O_2$	150.17
48	3-Hydroxyanthranilic acid	34.93	0.08	$C_7H_7NO_3$	153.14
49	2,5-Piperazinedione	36.12	0.05	$C_4H_6N_2O_2$	114.10
50	4-Aminobenzoic acid	36.83	0.20	$C_7H_7NO_2$	137.14
51	Indole-3-carboxylic acid	36.92	0.12	$C_9H_7NO_2$	161.16
52	Heneicosane	36.97	0.08	$C_{21}H_{44}$	296.57
53	Eicosane	36.97	0.08	$C_{20}H_{42}$	282.55
54	2-Mercaptobenzothiazole	37.32	0.38	$C_7H_5NS_2$	167.25
55	Hexadecanoic acid	37.88	3.07	$C_{16}H_{32}O_2$	256.42
56	Methyl stearate	39.42	0.15	$C_{19}H_{38}O_2$	298.50
57	1-Docosene	40.73	0.45	$C_{22}H_{44}$	308.59
58	9,12-Octadecadienoic acid	40.95	0.09	$C_{18}H_{32}O_2$	280.40
59	Androst-2-en-17-amine	41.04	0.13	$C_{19}H_{31}N$	273.50
60	trans-9-Octadecenoic acid	41.06	0.32	$C_{18}H_{34}O_2$	282.46
61	Octadecanoic acid	41.59	1.87	$C_{18}H_{36}O_2$	284.48
62	Mannopyranose	44.26	0.24	$C_6H_{12}O_6$	180.16
63	Octacosanol	44.28	0.36	$C_{28}H_{58}O$	410.76
64	Uridine	45.11	0.28	$C_9H_{12}N_2O_6$	244.20
65	2'-Deoxyadenosine	47.22	0.06	$C_{10}H_{13}N_5O_3$	251.24
66	1-Methyladenosine	47.45	1.61	$C_{11}H_{15}N_5O_4$	281.27

22

Phytochemical sub-classes identified in CSPL6. Data corresponds to Fig. 4A. Color shade (red to white) indicates a high to low level of abundance.

	Metabolite Set	Total	Hits	Expect	P value	Holm P	FDR
	Saturated Fatty Acids	38	9	0.0103	6.6E-25	4.82E-22	4.82E-22
	Hydrocarbons	52	6	0.0142	6.17E-15	4.5E-12	2.26E-12
	Alkanes	42	5	0.0114	1.05E-12	7.62E-10	2.55E-10
	Organic dicarboxylic acids	23	3	0.00626	3.37E-8	2.45E-5	6.15E-6
	Unsaturated Fatty Acids	267	4	0.0727	9.64E-7	7.01E-4	1.33E-4
	Straight chain Fatty Acids	6	2	0.00163	1.09E-6	7.92E-4	1.33E-4
	Dicarboxylic acids	140	3	0.0381	8.32E-6	0.00603	8.69E-4
	Pyridoxines	2	1	5.45E-4	5.45E-4	0.394	0.0442
	Tyrosols	2	1	5.45E-4	5.45E-4	0.394	0.0442
	Phenols	3	1	8.17E-4	8.17E-4	0.59	0.0498
	Aminopyrimidines	3	1	8.17E-4	8.17E-4	0.59	0.0498
	Benzenetriols	3	1	8.17E-4	8.17E-4	0.59	0.0498
	Cinnamic acids	4	1	0.00109	0.00109	0.783	0.0569
	Biopterins	4	1	0.00109	0.00109	0.783	0.0569
	Hydroxybenzoic acids	6	1	0.00163	0.00163	1.0	0.0746
	Pyrimidine ribonucleosides	6	1	0.00163	0.00163	1.0	0.0746
	Purine ribonucleosides	8	1	0.00218	0.00218	1.0	0.0808
	Quinoline carboxylic acids	8	1	0.00218	0.00218	1.0	0.0808
	Disaccharides	9	1	0.00245	0.00245	1.0	0.0808
	TCA acids	9	1	0.00245	0.00245	1.0	0.0808
	Phenethylamines	9	1	0.00245	0.00245	1.0	0.0808
	Benzyl alcohols	9	1	0.00245	0.00245	1.0	0.0808
	Hydroxy Fatty Acids	274	2	0.0746	0.0026	1.0	0.0808
	Amino acids	277	2	0.0754	0.00265	1.0	0.0808
	Sugar alcohols	12	1	0.00327	0.00326	1.0	0.0954
	Phenylacetic acids	15	1	0.00408	0.00408	1.0	0.115
	Monosaccharides	18	1	0.0049	0.00489	1.0	0.132
	Phenylpropanoic acids	23	1	0.00626	0.00625	1.0	0.163
	Benzoic acids	24	1	0.00654	0.00652	1.0	0.164
	Fatty alcohols	452	2	0.123	0.00686	1.0	0.167
	Oxo Fatty Acids	43	1	0.0117	0.0116	1.0	0.275
	Retinoids	70	1	0.0191	0.0189	1.0	0.431
	C21 steroids	148	1	0.0403	0.0395	1.0	0.861
	Oxo Fatty Acids	150	1	0.0408	0.04	1.0	0.861
	Branched Fatty Acids	372	1	0.101	0.0964	1.0	1.0

23

Table S4. Phytochemical sub-classes identified in CSPL5b. Data corresponds to Fig. 4B. Color shade (red to white) indicates a high to low level of abundance.

	Metabolite Set	Total	Hits	Expect	P value	Holm P	FDR
	Saturated Fatty Acids	38	8	0.00939	3.7E-22	2.71E-19	2.71E-19
	Alkanes	42	4	0.0104	3.66E-10	2.67E-7	1.34E-7
	Hydrocarbons	52	4	0.0128	8.83E-10	6.44E-7	2.15E-7
	Organic dicarboxylic acids	23	3	0.00568	2.5E-8	1.82E-5	4.57E-6
	Straight chain Fatty Acids	6	2	0.00148	8.97E-7	6.52E-4	1.31E-4
	Unsaturated aliphatic hydrocarbons	12	2	0.00297	3.94E-6	0.00286	4.8E-4
	Tyrosols	2	1	4.94E-4	4.94E-4	0.358	0.0493
	Dicarboxylic acids	140	2	0.0346	5.7E-4	0.412	0.0493
	Phenols	3	1	7.41E-4	7.41E-4	0.536	0.0493
	Aminopyrimidines	3	1	7.41E-4	7.41E-4	0.536	0.0493
	Benzenetriols	3	1	7.41E-4	7.41E-4	0.536	0.0493
	Indolecarboxylic acids	4	1	9.88E-4	9.88E-4	0.711	0.0602
	Purine deoxyribonucleosides	5	1	0.00124	0.00123	0.888	0.0645
	Medium-chain keto acids	5	1	0.00124	0.00123	0.888	0.0645
	Hydroxybenzoic acids	6	1	0.00148	0.00148	1.0	0.0677
	Pyrimidine ribonucleosides	6	1	0.00148	0.00148	1.0	0.0677
	Aminobenzoic acids	7	1	0.00173	0.00173	1.0	0.0743
	Purine ribonucleosides	8	1	0.00198	0.00198	1.0	0.0784
	Unsaturated Fatty Acids	267	2	0.066	0.00204	1.0	0.0784
	TCA acids	9	1	0.00222	0.00222	1.0	0.0812
	Sugar alcohols	12	1	0.00297	0.00296	1.0	0.103
	Pyridinecarboxylic acids	14	1	0.00346	0.00345	1.0	0.113
	Phenylacetic acids	15	1	0.00371	0.0037	1.0	0.113
	Indoles	15	1	0.00371	0.0037	1.0	0.113
	Monosaccharides	18	1	0.00445	0.00444	1.0	0.13
	Dipeptides	416	2	0.103	0.00484	1.0	0.136
	Phenylpropanoic acids	23	1	0.00568	0.00567	1.0	0.148
	Fatty alcohols	452	2	0.112	0.00568	1.0	0.148
	Benzoic acids	24	1	0.00593	0.00591	1.0	0.149
	Branched Fatty Acids	38	1	0.00939	0.00935	1.0	0.228
	Oxo Fatty Acids	43	1	0.0106	0.0106	1.0	0.249
	Fatty acid esters	133	1	0.0329	0.0323	1.0	0.739
	Oxo Fatty Acids	150	1	0.0371	0.0364	1.0	0.806
	Amino acids	277	1	0.0685	0.0662	1.0	1.0
	Unsaturated Fatty Acids	911	1	0.225	0.202	1.0	1.0
	Wax monoesters	949	1	0.235	0.21	1.0	1.0

24

Table S5. Metabolic pathway impact for CSPL6. Data corresponds to Fig. 4D.

Pathway Name	Match Status	p	-log(p)	Holm p	FDR	Impact
Biosynthesis of unsaturated fatty acids	4/22	2.2349E-4	3.6507	0.021455	0.021455	0.0
Butanoate metabolism	3/17	0.001686	2.7731	0.16017	0.080928	0.07143
Fatty acid biosynthesis	4/56	0.0080343	2.0951	0.75523	0.19366	0.01429
Glyoxylate and dicarboxylate metabolism	3/29	0.0080691	2.0932	0.75523	0.19366	0.09678
Phenylalanine metabolism	2/11	0.01066	1.9722	0.98072	0.20467	0.125
Sulfur metabolism	2/15	0.019633	1.707	1.0	0.31412	0.0
Propanoate metabolism	2/20	0.033971	1.4689	1.0	0.46588	0.04545
Synthesis and degradation of ketone bodies	1/4	0.058089	1.2359	1.0	0.61962	0.0
Linoleic acid metabolism	1/4	0.058089	1.2359	1.0	0.61962	0.66667
C5-Branched dibasic acid metabolism	1/6	0.085912	1.0659	1.0	0.82475	0.25
Aminoacyl-tRNA biosynthesis	2/46	0.14701	0.83264	1.0	1.0	0.07408
Vitamin B6 metabolism	1/11	0.15208	0.81793	1.0	1.0	0.14286
Nitrogen metabolism	1/12	0.16475	0.78317	1.0	1.0	0.0
Tyrosine metabolism	1/16	0.21366	0.67028	1.0	1.0	0.0
Cutin, suberine, and wax biosynthesis	1/18	0.23707	0.62512	1.0	1.0	0.07143
Citrate cycle (TCA cycle)	1/20	0.25982	0.58532	1.0	1.0	0.07143
Glycerolipid metabolism	1/21	0.27095	0.56711	1.0	1.0	0.03704
Alanine, aspartate and glutamate metabolism	1/22	0.28192	0.54987	1.0	1.0	0.0
Starch and sucrose metabolism	1/22	0.28192	0.54987	1.0	1.0	0.02857
Pyruvate metabolism	1/22	0.28192	0.54987	1.0	1.0	0.03448
Valine, leucine, and isoleucine biosynthesis	1/22	0.28192	0.54987	1.0	1.0	0.05
Thiamine metabolism	1/22	0.28192	0.54987	1.0	1.0	0.05263
Fatty acid elongation	1/23	0.29274	0.53352	1.0	1.0	0.0
Glycolysis / Gluconeogenesis	1/26	0.32425	0.48912	1.0	1.0	0.02941
Glutathione metabolism	1/26	0.32425	0.48912	1.0	1.0	0.0303
Galactose metabolism	1/27	0.33445	0.47566	1.0	1.0	0.0
Tryptophan metabolism	1/28	0.34451	0.4628	1.0	1.0	0.04348
alpha-Linolenic acid metabolism	1/28	0.34451	0.4628	1.0	1.0	0.08333
Cyanoamino acid metabolism	1/29	0.35442	0.45048	1.0	1.0	0.05
Glycine, serine and threonine metabolism	1/33	0.39265	0.40599	1.0	1.0	0.13514
Arginine and proline metabolism	1/34	0.40187	0.39592	1.0	1.0	0.05714
Valine, leucine and isoleucine degradation	1/37	0.42872	0.36783	1.0	1.0	0.0
Fatty acid degradation	1/37	0.42872	0.36783	1.0	1.0	0.02174
Pyrimidine metabolism	1/38	0.43741	0.35911	1.0	1.0	0.04

Table S6. Metabolic impact pathway for CSPL5b. Data corresponds to Fig. 4E.

Pathway Name	Match Status	p	-log(p)	Holm p	FDR	Impact
Butanoate metabolism	3/17	9.1919E-4	3.0366	0.088242	0.088242	0.07143
Biosynthesis of unsaturated fatty acids	3/22	0.0020038	2.6982	0.19036	0.09618	0.0
Fatty acid biosynthesis	4/56	0.0037623	2.4245	0.35366	0.12039	0.01429
Sulfur metabolism	2/15	0.013311	1.8758	1.0	0.31947	0.0
Propanoate metabolism	2/20	0.023237	1.6338	1.0	0.44558	0.04545
Pyruvate metabolism	2/22	0.027849	1.5552	1.0	0.44558	0.06896
Glycolysis / Gluconeogenesis	2/26	0.038075	1.4194	1.0	0.45812	0.05882
Glyoxylate and dicarboxylate metabolism	2/29	0.046555	1.332	1.0	0.45812	0.03226
Synthesis and degradation of ketone bodies	1/4	0.047721	1.3213	1.0	0.45812	0.0
Linoleic acid metabolism	1/4	0.047721	1.3213	1.0	0.45812	0.66667
Phenylalanine metabolism	1/11	0.1261	0.89929	1.0	1.0	0.0
Nicotinate and nicotinamide metabolism	1/13	0.14735	0.83166	1.0	1.0	0.0
Tyrosine metabolism	1/16	0.17831	0.74883	1.0	1.0	0.0
Cutin, suberine and wax biosynthesis	1/18	0.19836	0.70255	1.0	1.0	0.07143
Citrate cycle (TCA cycle)	1/20	0.21794	0.66166	1.0	1.0	0.07143
Glycerolipid metabolism	1/21	0.22756	0.6429	1.0	1.0	0.03704
Alanine, aspartate and glutamate metabolism	1/22	0.23707	0.62512	1.0	1.0	0.0
Fatty acid elongation	1/23	0.24647	0.60823	1.0	1.0	0.0
Galactose metabolism	1/27	0.28299	0.54823	1.0	1.0	0.0
Folate biosynthesis	1/27	0.28299	0.54823	1.0	1.0	0.03448
Tryptophan metabolism	1/28	0.29185	0.53484	1.0	1.0	0.04348
Valine, leucine and isoleucine degradation	1/37	0.3671	0.43521	1.0	1.0	0.0
Fatty acid degradation	1/37	0.3671	0.43521	1.0	1.0	0.02174
Pyrimidine metabolism	1/38	0.37498	0.42599	1.0	1.0	0.04
Purine metabolism	1/63	0.54434	0.26413	1.0	1.0	0.01333

Table S7. Bioactivities of identified compounds. ▲ means increase; ▼ means decrease; ● means no change.

Compounds	Bioactivities	References
**Palmitic acid (hexadecanoic acid)**	PLA_2 ▼ (enzyme kinetic study)	(Aparna et al., 2012)
**Phenylacetic acid, (Benzeneacetic acid)**	Aux/IAAs ▼, TIR1/AFB ▼	(Shimizu-Mitao & Kakimoto, 2014)
	Phytotoxin, Benzoic acid ▲	(Kawazu, Zhang, & Kanzaki, 1996)
	Antimicrobial activity	(Kim et al., 2004)
	Antifungal activity	(Hwang, Lim, Kim, Lee, & Moon, 2001; Slininger, Burkhead, & Schisler, 2004)
	Myeloperoxidase ▼, PGE_2▼, NFκB ▼, COX-2 ▼, and mast cell activation ▼	(K. M. Kwon et al., 2024)
**Succinic acid (Butanedioic acid)**	Antioxidant, protein SH groups ▼, ceruloplasmin ▲	(Bespyatykh, Kokorina, & Domskii, 2011)
	Cardiomyocyte Akt ▲	(Tang et al., 2013)
**Stearic acid (Octadecanoic acid)**	Lipid peroxidation ▼, PPAR-γ ▲, SOD ▲, CAT ▲	(Z.-j. Wang, Liang, Li, Yu, & Yin, 2007)
	NFκB ▼	(Pan et al., 2010)
**Tetradecanoic acid (myristic acid)**	Free radical scavenging ▲, COX ▼	(Henry, Momin, Nair, & Dewitt, 2002)
	Scavenger receptor BI ▼, cholesteryl ester ▼, sterol 27 hydroxylase ▲, 3-hydroxy-3-methyl glutaryl coenzyme A reductase ●	(Loison, Mendy, Sérougne, & Lutton, 2002)
	Modulation ▼▲ of IL-1, TNF-α, IL-8, p38, JNK kinases	(Håversen, Danielsson, Fogelstrand, & Wiklund, 2009)
**Propionic acid (Propanoic acid)**	Leptin mRNA expression ▲	(S. a. H. Al-Lahham et al., 2010)
	Lipoprotein lipase ▲, TNF-α ▼, IL-4 ▼, IL-10 ▼, IL-8 ▼, MIP-1α ▼, MIP-1β ▼, CCL5 ▼, and CXCL10 ▼	(S. a. Al-Lahham et al., 2012)
	T-cell numbers ▲, IL-10 ▲, and NPC1|1 ▼	(Haghikia et al., 2022)
	Gluconeogenic enzymes ▼; AMPK ▲	(Yoshida, Ishii, & Akagawa, 2019)
**Butyrate (Butanoic acid)**	IL-6 ▼, IL-1β ▼, and IL-10 ▲	(F. Wang et al., 2017)
	Cholesterol absorption ▼, NPC1l1 ▼, ABCG5 ▲,	(Y. Chen et al., 2018)

	and ABCG8 ▲	
	AKT ▲, and NF-κB signaling ▲	(He et al., 2020)
	IL-22 ▲ by $CD4^+$ T cells and ILCs	(Yang et al., 2020)
**Tryptophol (3-(2-Hydroxyethyl)indole)**	Anti-inflammatory properties, IFN- γ ▼, TNF-α ▼	(Schirmer et al., 2016)
**3-Hydroxyanthranilic acid**	TNF-α ▼, IP-10 ▼, IL-8 ●, HO-1 ▼	(Krause et al., 2011)
	inhibits the PI3K/Akt/mTOR and NF-κB signaling pathways, IL-6 ▼, and TNF-α ▼	(K. Lee, Kwak, & Pyo, 2016)
**Cinnamic acid**	Obesity ▼, epididymal fat accumulation ▼, insulin resistance ▼, glucose intolerance ▼, dyslipidemia ▼<br><br>TNF-α ▼, and macrophage infiltration ▼<br>Reduced $Ly6c^{high}$ monocytes, M1 adipose tissue macrophages, and hypothalamus microglial activation	(A. G. Lee, Kang, Im, & Pak, 2022)
	TNF-α ▼, IL-6 ▼, MPO ▼, and TLR-4 ▼	(Rezaei et al., 2024)
**Isotretinoin (13-cis-Retinoic acid)**	Antimicrobial activity against *Propionibacterium acnes*	(Raza et al., 2013)
**Camphoric acid**	Antifungal activity against *Physalospora piricola*	(Ma et al., 2014)
**Phloroglucinol**	Inhibits VEGF-dependent migration, EPCs, tumor growth, angiogenesis, and CD45(-)/CD34(+) progenitor mobilization	(Y.-H. Kwon et al., 2012)
	iNOS▼, and NF-κB ▼	(Li, Khan, Qiu, & Li, 2018)
	ROS ▼, NF-κB ▼, Bcl-2 ▼, MMP-2 ▼, VEGF ▼; TNF-α ▼, IL-6 ▼, IL-1β ▼, and NO ▼	(Veena, Popavath, Kennedy, & Sakthivel, 2015)
**Quinaldic acid (2-Quinolinecarboxylic acid)**	Anti-microbial agent: ▼ *Clostridium difficile* and *C. perfringens*, *C. difficile*, and *C. perfringens*	(C.-H. Lee & Lee, 2009)
**Valproate (Valproic acid)**	TNF-α ▼, leukocyte infiltration ▼, and MPO ▼	(Ximenes et al., 2013)
	Inflammatory proteins ▼ (pNFκB, iNOS, and COX-2), pro-apoptotic proteins ▼ (pAKT/AKT and pGSK-3β/GSK-3β), and proinflammatory cytokines ▼ (TNF-α and IL-1β)	(J.-Y. Chen et al., 2018)
**Azelaic acid**	IL-1β ▼, IL-6 ▼, TNF-α ▼, and NF-κB ▼	(Mastrofrancesco et al., 2010)
	Antioxidant and free radical scavengers	(Passi et al., 1991; Spaggiari et al., 2023)

PLA_2= Phospholipase A_2; **Aux=** Auxin; **IAA=** Indole-3-acetic acid; **TIR1=** Transport inhibitor response 1; **AFB=** Auxin signaling F-box; **PGE_2=** Prostaglandin E_2; **NF-κβ=** Nuclear factor kappa beta; **COX=** Cyclooxygenase; **PPAR-γ=** peroxisome proliferator-activated receptor γ; **SOD=** Superoxide dismutase; **CAT=** Catalase; **IL=** Interleukin; **TNF-α=** Tumor necrosis factor alpha; **JNK=** Jun N-terminal kinase; **MIP=**

macrophage inflammatory protein; **CCL5**=;chemokine (C-C motif) ligand 5; **CXCL10**= C-X-C motif chemokine ligand 10; **NPC1l1=** Niemann-Pick C1-like 1; **AMPK=** AMP-activated protein kinase; **ABCG5=** ATP-binding cassette transporters G5; **ABCG8=** ATP-binding cassette transporters G8; **ILCs=** Innate lymphoid cells; **IFN-γ**= Interferon gamma; **IP-10=** interferon gamma-induced protein 10; **HO-1=** Heme oxygenase 1; **PI3K=** phosphoinositide-3-kinase; **mTOR=** mammalian target of rapamycin; **MPO=** Myeloperoxidase; **TLR4=** Toll-like receptor 4; **iNOS**= Inducible nitric oxide synthase; **ROS**= Reactive oxygen species; **Bcl-2=** B-cell lymphoma 2; **MMP-2=** matrix metalloproteinase-2; **EPCs=** endothelial progenitor cells; **VEGF=** vascular endothelial growth factor; **NO**= Nitric oxide; **GSK-3=** glycogen synthase kinase-3

References

Al-Lahham, S. a., Roelofsen, H., Rezaee, F., Weening, D., Hoek, A., Vonk, R., & Venema, K. (2012). Propionic acid affects immune status and metabolism in adipose tissue from overweight subjects. *European journal of clinical investigation, 42*(4), 357-364.

Al-Lahham, S. a. H., Roelofsen, H., Priebe, M., Weening, D., Dijkstra, M., Hoek, A., . . . Vonk, R. J. (2010). Regulation of adipokine production in human adipose tissue by propionic acid. *European journal of clinical investigation, 40*(5), 401-407.

Aparna, V., Dileep, K. V., Mandal, P. K., Karthe, P., Sadasivan, C., & Haridas, M. (2012). Anti-inflammatory property of n-hexadecanoic acid: structural evidence and kinetic assessment. *Chemical biology & drug design, 80*(3), 434-439.

Bespyatykh, O. Y., Kokorina, A., & Domskii, I. (2011). State of antioxidant system of furbearers after injection of succinic acid. *Russian agricultural sciences, 37*(6), 516-519.

Chen, J.-Y., Chu, L.-W., Cheng, K.-I., Hsieh, S.-L., Juan, Y.-S., & Wu, B.-N. (2018). Valproate reduces neuroinflammation and neuronal death in a rat chronic constriction injury model. *Scientific reports, 8*(1), 16457.

Chen, Y., Xu, C., Huang, R., Song, J., Li, D., & Xia, M. (2018). Butyrate from pectin fermentation inhibits intestinal cholesterol absorption and attenuates atherosclerosis in apolipoprotein E-deficient mice. *The Journal of nutritional biochemistry, 56*, 175-182.

Haghikia, A., Zimmermann, F., Schumann, P., Jasina, A., Roessler, J., Schmidt, D., . . . Aigner, A. (2022). Propionate attenuates atherosclerosis by immune-dependent regulation of intestinal cholesterol metabolism. *European heart journal, 43*(6), 518-533.

Håversen, L., Danielsson, K. N., Fogelstrand, L., & Wiklund, O. (2009). Induction of proinflammatory cytokines by long-chain saturated fatty acids in human macrophages. *Atherosclerosis, 202*(2), 382-393.

He, Q., Han, C., Huang, L., Yang, H., Hu, J., Chen, H., . . . Lin, H. (2020). Astragaloside IV alleviates mouse slow transit constipation by modulating gut microbiota profile and promoting butyric acid generation. *Journal of Cellular and Molecular Medicine, 24*(16), 9349-9361.

Henry, G. E., Momin, R. A., Nair, M. G., & Dewitt, D. L. (2002). Antioxidant and cyclooxygenase activities of fatty acids found in food. *Journal of agricultural and food chemistry, 50*(8), 2231-2234.

Hwang, B. K., Lim, S. W., Kim, B. S., Lee, J. Y., & Moon, S. S. (2001). Isolation and in vivo and in vitro antifungal activity of phenylacetic acid and sodium phenylacetate from Streptomyces humidus. *Applied and Environmental Microbiology, 67*(8), 3739-3745.

Kawazu, K., Zhang, H., & Kanzaki, H. (1996). Accumulation of benzoic acid in suspension cultured cells of Pinus thunbergii Parl. in response to phenylacetic acid administration. *Bioscience, biotechnology, and biochemistry, 60*(9), 1410-1412.

Kim, Y., Cho, J.-Y., Kuk, J.-H., Moon, J.-H., Cho, J.-I., Kim, Y.-C., & Park, K.-H. (2004). Identification and antimicrobial activity of phenylacetic acid produced by Bacillus licheniformis isolated from fermented soybean, Chungkook-Jang. *Current microbiology, 48*, 312-317.

Krause, D., Suh, H.-S., Tarassishin, L., Cui, Q. L., Durafourt, B. A., Choi, N., . . . Zhao, M.-L. (2011). The tryptophan metabolite 3-hydroxyanthranilic acid plays anti-inflammatory and neuroprotective roles during inflammation: role of hemeoxygenase-1. *The American journal of pathology, 179*(3), 1360-1372.

Kwon, K. M., Kim, E.-H., Sim, K. H., Lee, Y. J., Kang, E.-J., Han, K.-H., . . . Hwang, I. H. (2024). Phenylacetic acid, an anti-vaginitis metabolite produced by the vaginal symbiotic bacterium Chryseobacterium gleum. *Scientific reports, 14*(1), 12226.

Kwon, Y.-H., Jung, S.-Y., Kim, J.-W., Lee, S.-H., Lee, J.-H., Lee, B.-Y., & Kwon, S.-M. (2012). Phloroglucinol inhibits the bioactivities of endothelial progenitor cells and suppresses tumor angiogenesis in LLC-tumor-bearing mice. *PLoS One, 7*(4), e33618.

Lee, A. G., Kang, S., Im, S., & Pak, Y. K. (2022). Cinnamic acid attenuates peripheral and hypothalamic inflammation in high-fat diet-induced obese mice. *Pharmaceutics, 14*(8), 1675.

Lee, C.-H., & Lee, H.-S. (2009). Growth inhibiting activity of quinaldic acid isolated from Ephedra pachyclada against intestinal bacteria. *Journal of the Korean Society for Applied Biological Chemistry, 52*, 331-335.

Lee, K., Kwak, J.-H., & Pyo, S. (2016). Inhibition of LPS-induced inflammatory mediators by 3-hydroxyanthranilic acid in macrophages through suppression of PI3K/NF-κB signaling pathways. *Food & function, 7*(7), 3073-3082.

Li, N., Khan, S. I., Qiu, S., & Li, X.-C. (2018). Synthesis and anti-inflammatory activities of phloroglucinol-based derivatives. *Molecules, 23*(12), 3232.

Loison, C., Mendy, F., Sérougne, C., & Lutton, C. (2002). Dietary myristic acid modifies the HDL-cholesterol concentration and liver scavenger receptor BI expression in the hamster. *British Journal of Nutrition, 87*(3), 199-210.

Ma, X.-L., Li, F.-Y., Duan, W.-G., Liao, J.-N., Lin, Z.-d., Lin, G.-S., . . . Lei, F.-H. (2014). Synthesis and antifungal activity of camphoric acid-based acylhydrazone compounds. *Holzforschung, 68*(8), 889-895.

Mastrofrancesco, A., Ottaviani, M., Aspite, N., Cardinali, G., Izzo, E., Graupe, K., . . . Picardo, M. (2010). Azelaic acid modulates the inflammatory response in normal human keratinocytes through PPARγ activation. *Experimental dermatology, 19*(9), 813-820.

Pan, P.-H., Lin, S.-Y., Ou, Y.-C., Chen, W.-Y., Chuang, Y.-H., Yen, Y.-J., . . . Chen, C.-J. (2010). Stearic acid attenuates cholestasis-induced liver injury. *Biochemical and biophysical research communications, 391*(3), 1537-1542.

Passi, S., Picardo, M., Zompetta, C., Luca, C. d., Breathnach, A. S., & Nazzaro-porro, M. (1991). The oxyradical-scavenging activity of azelaic acid in biological systems. *Free radical research communications, 15*(1), 17-28.

Raza, K., Singh, B., Singla, S., Wadhwa, S., Garg, B., Chhibber, S., & Katare, O. P. (2013). Nanocolloidal carriers of isotretinoin: antimicrobial activity against Propionibacterium acnes and dermatokinetic modeling. *Molecular pharmaceutics, 10*(5), 1958-1963.

Rezaei, Z., Momtaz, S., Gharazi, P., Rahimifard, M., Baeeri, M., Abdollahi, A. R., . . . Farzaei, M. H. (2024). Cinnamic Acid Ameliorates Acetic Acid-induced Inflammatory Response through Inhibition of TLR-4 in Colitis Rat Model. *Anti-Inflammatory & Anti-Allergy Agents in Medicinal Chemistry (Formerly Current Medicinal Chemistry-Anti-Inflammatory and Anti-Allergy Agents), 23*(1), 21-30.

Schirmer, M., Smeekens, S. P., Vlamakis, H., Jaeger, M., Oosting, M., Franzosa, E. A., . . . Bonder, M. J. (2016). Linking the human gut microbiome to inflammatory cytokine production capacity. *Cell, 167*(4), 1125-1136. e1128.

Shimizu-Mitao, Y., & Kakimoto, T. (2014). Auxin sensitivities of all Arabidopsis Aux/IAAs for degradation in the presence of every TIR1/AFB. *Plant and Cell Physiology, 55*(8), 1450-1459.

Slininger, P., Burkhead, K., & Schisler, D. (2004). Antifungal and sprout regulatory bioactivities of phenylacetic acid, indole-3-acetic acid, and tyrosol isolated from the potato dry rot suppressive bacterium Enterobacter cloacae S11: T: 07. *Journal of Industrial Microbiology and Biotechnology, 31*(11), 517-524.

Spaggiari, C., Annunziato, G., Spadini, C., Montanaro, S. L., Iannarelli, M., Cabassi, C. S., & Costantino, G. (2023). Extraction and quantification of azelaic acid from different wheat samples (triticum durum desf.) and evaluation of their antimicrobial and antioxidant activities. *Molecules, 28*(5), 2134.

Tang, X.-L., Liu, J.-X., Li, P., Dong, W., Li, L., Zheng, Y.-Q., & Hou, J.-C. (2013). Protective effect of succinic acid on primary cardiomyocyte hypoxia/reoxygenation injury. *Zhongguo Zhong yao za zhi= Zhongguo Zhongyao Zazhi= China Journal of Chinese Materia Medica, 38*(21), 3742-3746.

Veena, V. K., Popavath, R. N., Kennedy, K., & Sakthivel, N. (2015). In vitro antiproliferative, pro-apoptotic, antimetastatic and anti-inflammatory potential of 2, 4-diacteylphloroglucinol (DAPG) by Pseudomonas aeruginosa strain FP10. *Apoptosis, 20*, 1281-1295.

Wang, F., Liu, J., Weng, T., Shen, K., Chen, Z., Yu, Y., . . . Jin, S. (2017). The inflammation induced by lipopolysaccharide can be mitigated by short-chain fatty acid, butyrate, through upregulation of IL-10 in septic shock. *Scandinavian journal of immunology, 85*(4), 258-263.

Wang, Z.-j., Liang, C.-l., Li, G.-m., Yu, C.-y., & Yin, M. (2007). Stearic acid protects primary cultured cortical neurons against oxidative stress. *Acta Pharmacologica Sinica, 28*(3), 315-326.

Ximenes, J. C. M., de Oliveira Gonçalves, D., Siqueira, R. M. P., Neves, K. R. T., Santos Cerqueira, G., Correia, A. O., . . . da Graça Naffah-Mazzacorati, M. (2013). Valproic acid: an anticonvulsant drug with potent antinociceptive and anti-inflammatory properties. *Naunyn-Schmiedeberg's archives of pharmacology, 386*, 575-587.

Yang, W., Yu, T., Huang, X., Bilotta, A. J., Xu, L., Lu, Y., . . . Zhang, W. (2020). Intestinal microbiota-derived short-chain fatty acids regulation of immune cell IL-22 production and gut immunity. *Nature communications, 11*(1), 4457.

Yoshida, H., Ishii, M., & Akagawa, M. (2019). Propionate suppresses hepatic gluconeogenesis via GPR43/AMPK signaling pathway. *Archives of biochemistry and biophysics, 672*, 108057.
